# Novel role for receptor dimerization in post-translational processing and turnover of the GRα

**DOI:** 10.1038/s41598-018-32440-z

**Published:** 2018-09-24

**Authors:** Legh Wilkinson, Nicolette Verhoog, Ann Louw

**Affiliations:** Department of Biochemistry, Stellenbosch University, Stellenbosch, South Africa

## Abstract

Glucocorticoids (GCs), acting via the glucocorticoid receptor (GRα), remain the mainstay therapeutic choice for the treatment of inflammation. However, chronic GC use, aside from generating undesirable side-effects, results in GRα down-regulation, often coupled to a decrease in GC-responsiveness, which may culminate in acquired GC resistance. The current study presents evidence for a novel role of the dimerization state of the GRα in mediating GC-mediated GRα turnover. Through comparing the effects of dimerization promoting GCs on down-regulation of a transfected human wild type GRα (hGRwt) or a dimerization deficient GRα mutant (hGRdim), we established that a loss of receptor dimerization restricts GRα turnover, which was supported by the use of the dimerization abrogating Compound A (CpdA), in cells containing endogenous GRα. Moreover, we showed that the dimerization state of the GRα influenced the post-translational processing of the receptor, specifically hyper-phosphorylation at Ser404, which influenced the interaction of GRα with the E3 ligase, FBXW7α, thus hampering receptor turnover via the proteasome. Lastly, the restorative effects of CpdA on the GRα pool, in the presence of Dex, were demonstrated in a combinatorial treatment protocol. These results expand our understanding of factors that contribute to GC-resistance and may be exploited clinically.

## Introduction

Synthetic glucocorticoids (GCs) continue to be the preferred therapeutics for the treatment of diseases associated with chronic inflammation^1,2^, despite two major limitations, namely, the generation of undesirable side effects and the development of resistance to GC treatment^3–6^.

Over recent years, research has focused on investigating and developing selective glucocorticoid receptor modulators (SGRMs), which in essence aim to maintain a potent anti-inflammatory potential whilst having an improved side effect profile, by preferentially triggering the transrepression, rather than the transactivation, function of the glucocorticoid receptor α (GRα)^7^. Whilst SGRMs are proving somewhat successful in curbing the generation of undesirable side-effects^7–10^, there is still the lingering issue of developing acquired resistance to GC treatment following prolonged GC use.

GC resistance is an ever increasing threat, with approximately a third of all patients, receiving GC treatment, displaying a degree of insensitivity to treatment^11,12^. Specifically, 4–10% of asthma patients, 30% of rheumatoid arthritis patients, almost all chronic obstructive pulmonary disease (COPD) and sepsis patients^11^ and 10–30% of untreated acute lymphoblastic leukaemia (ALL) patients^13^ experience varying degrees of GC insensitivity. Many studies have demonstrated a direct correlation between the ability of a patient to respond to GC treatment and the amount of functional GRα protein available^14–16^. In short, disruptions in GRα function, are known to modulate the subcellular localization, ligand binding, and transactivation ability of the receptor, and are regulated by, amongst others, increases in additional GR isoforms (GRβ and GRγ) due to alternative splicing events, inactivating GRα mutations, the inflammatory cytokine profile of the cellular microenvironment and mutations/polymorphisms in the ERK pathway, as eloquently reviewed by Nicolaides *et al*.^17^, Oakley *et al*.^6,18^, and Patel *et al*.^19^. In some cases GC resistance is inherited and is frequently associated with alterations at the level of GRα function, caused by inactivating mutations^17^. However, more common is the development of an acquired GC resistance, often linked to disease-progression^20–22^ or prolonged GC treatment^23–25^, which reduces the level of the GR protein pool^26^.

The level of the GRα protein pool is maintained by two opposing cellular processes namely, synthesis and degradation. Both of these processes are governed by a number of GC-independent and GC-dependent molecular mechanisms, which control their rates and ultimately the level of the GRα protein pool.

Although GC-mediated regulation has been noted at the level of synthesis^27–29^, it is well documented that GC-mediated regulation of the GRα protein pool occurs at the level of protein degradation, specifically via the ubiquitin-proteasome system (UPS)^30,31^. The UPS, consists of a large number of components, which function collectively to ensure its highly specific nature^32^. In general, proteins are tagged through covalent post-translational modifications (PTMs), which provide the signal for recognition by the catalytic proteasome that subsequently mediates the degradation of the protein substrates^32^.

Both unliganded and liganded GRα undergoes a number of PTMs including phosphorylation and ubiquitination^18,33,34^, which are particularly important for the UPS. The GRα protein undergoes GC-mediated phosphorylation, at several identified serine or threonine^35,36^ residues. One of the more recently discovered sites of GRα phosphorylation in humans is Ser404, which is mediated, in a GC-dependent manner, by the glycogen synthase kinase 3β (GSK3β)^37^. This GC-mediated hyper-phosphorylation at Ser404 has implications in acquired GC resistance, as it has been shown to decrease overall GRα stability^37^.

Often, PTMs occur in a sequential manner and the PTM, ubiquitination, is thought to depend on preceding protein phosphorylation^32^. Ubiquitination of the GRα occurs at a single site, K419 in humans, which falls within the PEST motif ^31^. Interestingly, the K419 site occurs only slightly upstream from the phosphorylation site at S404. Wallace *et al*.^30,31^, elegantly demonstrated that mutation of K419 results in the abrogation of ligand-induced GRα protein down-regulation via the proteasome.

Unlike phosphorylation, the process of ubiquitination involves a number of enzymes, with a hierarchy of specificity for a protein, which function in a highly coordinated manner to generate a poly-ubiquitin chain^38,39^. Generally, E1 activating enzymes activate ubiquitin; where after the active ubiquitin is transferred to E2 conjugating enzymes. Subsequently, a specific E3 ligase bound to the protein substrate mediates the transfer of the activated ubiquitin molecule to the substrate. This process is repeated to form a poly-ubiquitin chain recognized by the proteasome, which then mediates protein degradation. A number of UPS enzymes, known to interact with the GRα protein in both a GC-independent and GC-dependent manner, have been identified^18,40–44^. Specifically, the inactive E2 conjugating enzyme, susceptibility gene 101 (TSG101)^41^, and the active E2 conjugating enzyme, ubiquitin-conjugating enzyme 7 (UbcH7)^42^, have been shown to influence GRα turnover. Additionally the E3 ligases, carboxy-terminus of heat shock protein 70-interacting protein (CHIP)^40^, murine (Mdm2) or human (Hdm2) double minute 2^43,45,46^, and F-box/WD repeat-containing protein 7 (FBXW7α)^37,44^, have all been implicated in GC-mediated GRα turnover. Hyper-phosphorylation GRα at S404 has been shown to regulate binding of FBXW7α^44^.

Both endogenous and exogenous GCs, such as the potent synthetic GC dexamethasone (Dex), are known to mediate GRα mRNA^28,47–50^ and protein^30,31,36,37,49^ turnover, resulting in a robust reduction in the GRα protein pool and subsequently driving the development of acquired GC resistance. In stark contrast, a number of studies have shown that CpdA, a SGRM, does not result in GRα down-regulation at either the mRNA or protein level^36,49,51^. Furthermore, following treatment with CpdA, the GRα protein is reported to have a half-life similar to that of the unliganded GRα protein^36^. Interestingly, CpdA does not result in GRα dimerization, in contrast to Dex treatment^52,53^, which hints at a possible role for ligand-induced GRα dimerization in ligand-induced GRα protein turnover.

These thought provoking effects of CpdA treatment on GRα dimerization and GRα down-regulation provided a concrete platform for the current study. Specifically, in the current study we present data demonstrating that the dimerization state of the GRα influences post-translational processing of the receptor, interaction with components of the UPS and subsequent degradation via the proteasome.

## Results

### Rate of hGRwt protein turnover is altered in a ligand-selective manner

**Figure 1:**
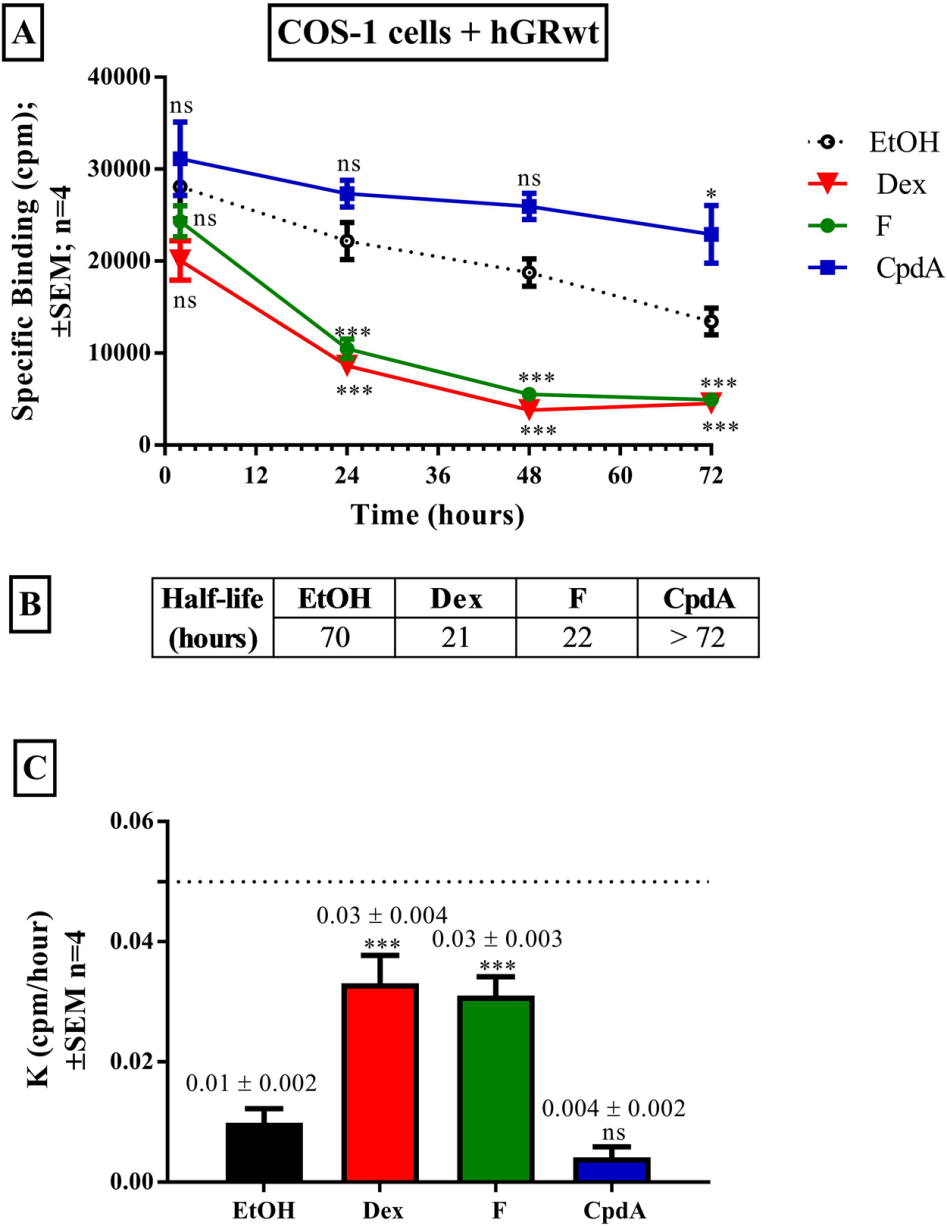
Rate of hGRwt protein turnover is altered in a ligand-selective manner. COS-1 cells were seeded into a 24 well plate (5 × 10^4^ cells/well) and transiently transfected the next day with hGRwt. Following 24 hours incubation, cells were treated with solvent (EtOH) or the GCs, Dex and F, or CpdA (10^−5^ M) for 2 to 72 hours. Thereafter, whole cell GRα-binding (**A**) was conducted using 20 nM [^3^H]-Dex. Once lysed, hGRwt levels were detected via a scintillation counter and specific binding values (cpm) were plotted against time. Specific binding values of lysates from solvent (EtOH) and treated cells were plotted for each time point. Whole cell GRα-binding results shown are representative of four independent experiments (average ± SEM), conducted in triplicate. Statistical analysis of logarithmically transformed data was conducted using a two-way ANOVA followed by a Bonferroni multiple comparisons post-test comparing each time point to solvent (EtOH) (ns, P > 0.05, *P < 0.05, **P < 0.01, ***P < 0.001). Half-lives (**B**) and rate constants (**C**, actual values above bars) were calculated using non-linear regression one-phase dissociation decay analysis. For statistical analysis of rate constants, one-way ANOVA followed by a Dunnett’s multiple comparisons post-test was conducted on logarithmically transformed data comparing K (cpm/hour) values to solvent (EtOH) (ns, P > 0.05, ***P < 0.001).

Although down-regulation of the unliganded GRα protein occurs, treatment with dimerization promoting GCs^54,55^, such as Dex and cortisol (F), increases the extent of turnover of the GRα^30,47,56,57^. In contrast, treatment with the dimerization abrogating CpdA^52,53^ induces minimal GRα turnover^36,49,51^. To quantitatively determine differences in unliganded and liganded GRα protein turnover, half-lives (t½) and turnover rate constants (K) were calculated per treatment condition. Specifically, COS-1 cells transiently transfected with hGRwt, were treated with solvent (EtOH), Dex, F or CpdA, for 2 to 72 hours. Unliganded hGRwt protein was degraded in a time-dependent manner (Fig. 1A) and has a half-life of 70 hours (Fig. 1B). Furthermore, the dimerization promoting GCs, Dex and F, significantly (P < 0.01) increased receptor turnover (Fig. 1A), resulting in a decreased half-life of hGRwt of 21 and 22 hours, respectively (Fig. 1B). For both GCs, maximal hGRwt protein down-regulation was reached following 48 hours of treatment. Interestingly, although no significant differences in the efficacies of Dex and F to mediate GRα turnover was noted, their potencies are significantly (P < 0.01) different (Supplementary Fig. 1). Moreover, it is clear that hGRwt protein turnover will occur at physiologically relevant GC concentrations (Supplementary Fig. 1). Specifically, free plasma F levels range from 10 nM to 50 nM during the circadian cycle in unstressed individuals^58,59^, which corresponds to a 15–39% reduction in the hGRwt pool, while the free plasma Dex levels range from 1–20 nM following a low dose administration^60^, which correlates with a 43–60% reduction in hGRwt levels.

Unlike for the dimerization promoting GCs, Dex and F, hGRwt protein turnover seemed to be virtually absent following treatment with the dimerization abrogating SGRM, CpdA (Fig. 1A). This difference is reflected in the rate constants for turnover, which indicated a significant (P < 0.001) increase in the rate of hGRwt protein turnover, from 0.01 cpm/hour for the unliganded receptor, to 0.03 cpm/hour following Dex and F treatment, while, in contrast, a substantial decrease in the rate of hGRwt protein turnover was observed following CpdA treatment (0.004 cpm/hour) (Fig. 1C). CpdA, unlike the dimerization promoting GCs, clearly exerts a restorative effect on the GRα protein pool.

### GRα dimerization is required for turnover of the GRα protein

The ability of CpdA to restrict GRα protein turnover^36,49,51^, combined with its capacity to prevent or even abrogate^52,53^ GRα dimerization, sparked our interest in a possible role for GRα dimerization in mediating ligand-induced turnover of the GRα protein. Thus, to determine the effects of a gain or loss of GRα dimerization on GRα protein turnover, the levels of hGRwt and hGRdim, a dimerization deficient mutant^61^, were compared in COS-1 cells, in the presence of dimerization promoting (Dex or F)^54,55^ or abrogating (CpdA)^51,52^ GRα ligands. Following which the effect was investigated in HepG2 cells, containing endogenous human GRα (hGRα).

**Figure 2:**
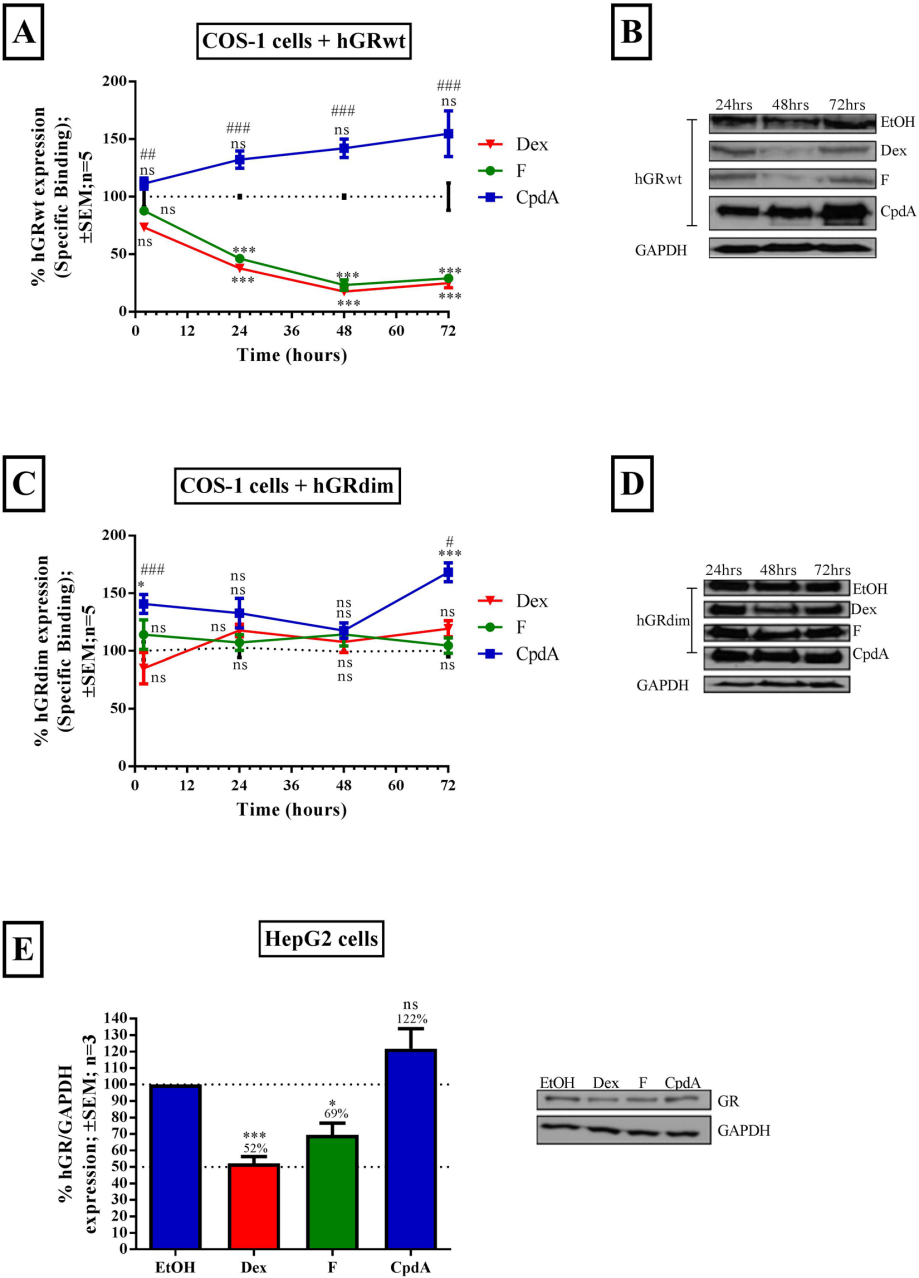
GRα dimerization is required for ligand-induced GRα protein turnover. COS-1 cells were seeded into a 24 well plate (5 × 10^4^ cells/well) and transiently transfected the next day with hGRwt (**A** and **B**) or hGRdim (**C** and **D**). Following 24 hours incubation, cells were treated with Dex, F or CpdA (10^−5^ M) for 2 to 72 hours. Thereafter hGRwt (**A**) and hGRdim (**C**) levels were monitored via whole cell GRα-binding, using 20 nM [^3^H]-Dex. Specific binding of lysates from solvent (EtOH) treated cells was set at 100% (dotted line) for each time point and % specific binding of lysates from compound treated cells were then determined relative to solvent (EtOH), at each time point, and plotted. Whole cell GRα-binding results shown are representative of five independent experiments (average ± SEM), conducted in triplicate. Statistical analysis of logarithmically transformed data for (**A** and **C**) was conducted using a two-way ANOVA followed by a Bonferroni multiple comparisons post-test comparing experimental values to solvent (EtOH) (ns, P > 0.05, **P < 0.01, ***P < 0.001) or to Dex (^#^P < 0.05, ^##^P < 0.01, ^###^P < 0.001). GRα protein levels of hGRwt (**B**) and hGRdim (**D**) were confirmed by Western blotting. The 24 hour treatment time point was repeated, with all ligands in the HepG2 cells (containing endogenous hGRα), which were seeded into a 12 well plate (5 × 10^4^ cell/well). GRα levels were assessed using Western blotting where GAPDH was probed to ensure equal protein loading. Western blots shown are representative of three independent experiments (**E**, inset). For quantification (**E**), the intensity of the hGRα and GAPDH bands was determined using UNSCANIT and hGRα levels were then normalized to GAPDH levels and expressed as a percentage (average ± SEM) of hGRα levels in the presence of the solvent (EtOH), which was set at 100% (dotted line). Statistical analysis of logarithmically transformed data for (**E**) was conducted using a one-way ANOVA with Dunnett’s multiple comparisons post-test comparing experimental values to solvent (EtOH) (ns, P > 0.05, *P < 0.05, ***P < 0.001). Full-length blots are presented in Supplementary Figure S6.

As expected gain of hGRwt dimerization via the dimerization promoting GCs Dex and F, significantly (P < 0.001) reduced the transiently transfected hGRwt protein pool over time (Fig. 2A and B) as well as the endogenous hGRα protein pool (P < 0.05) (Fig. 2E). Specifically already at 24 hours, Dex and F treatment reduced the pool of transiently transfected hGRwt to a mere 38 and 46% of the unliganded GRα (100%), respectively (Fig. 2A and B), while similarly, but slightly less robustly, endogenous hGRα protein levels in HepG2 cells were reduced to 52% and 69%, respectively (Fig. 2E). Unlike with hGRwt, loss of GRα dimerization through the use of hGRdim, restricted the GRα down-regulation induced by the dimerization promoting GCs, Dex and F, with the level of the hGRdim pool maintained over the time course (Fig. 2C and D). In support of the hGRdim result, loss of dimerization through CpdA treatment of the transfected hGRwt (Fig. 2A and B) and the endogenous hGRα (Fig. 2E), did not result in substantial reductions in the receptor protein pool.

To our knowledge, this is the first time that the ability of the hGRdim to undergo ligand-induced down-regulation has been investigated and collectively the results indicate a novel role for the dimerization state of the GRα protein as an important determinant for efficient ligand-induced turnover.

### Ligand-induced turnover of the GRα protein occurs predominantly via the proteasome

**Figure 3:**
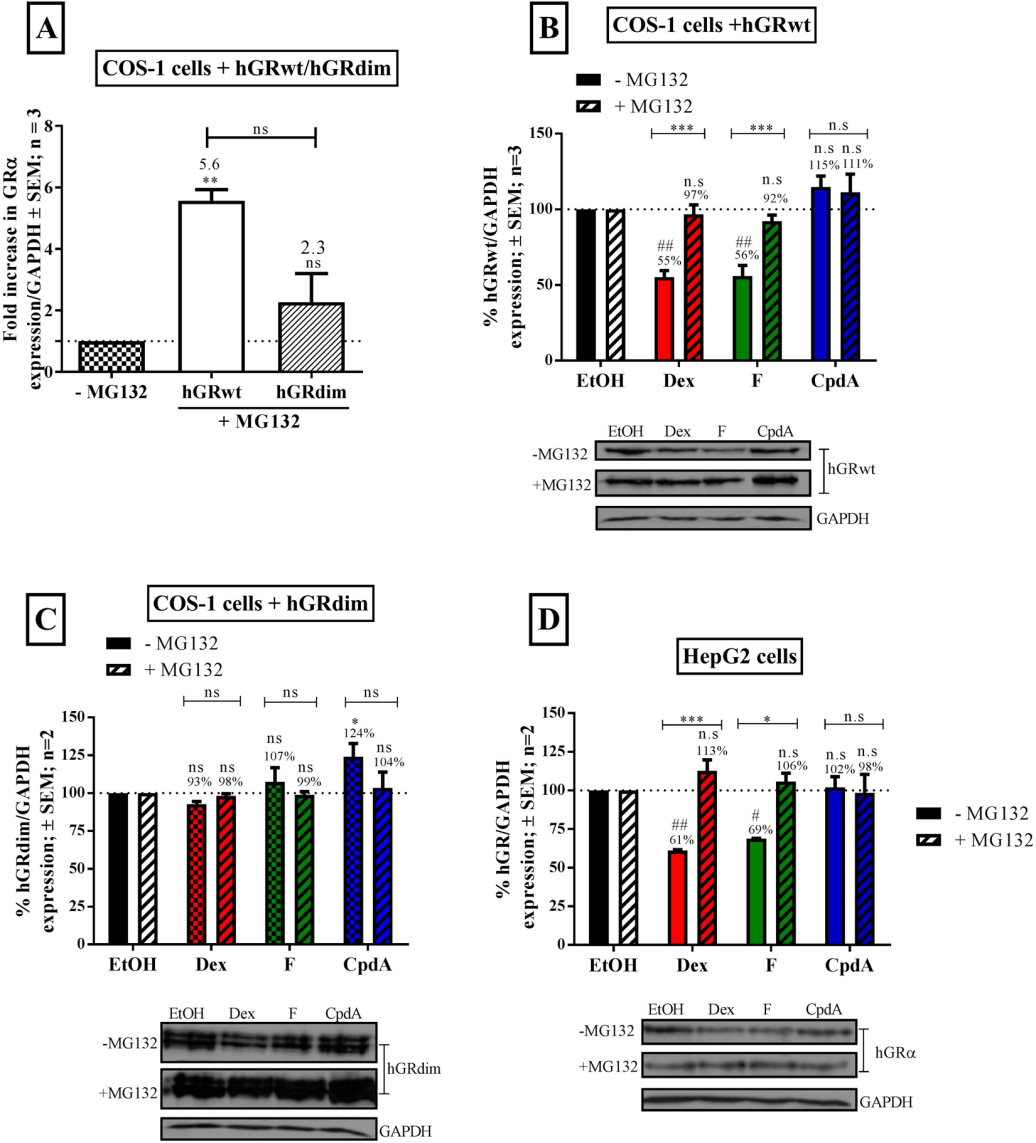
Ligand-induced GRα protein turnover occurs predominantly via the proteasome. COS-1 cells were seeded in a 12 well plate (5 × 10^4^ cell/well) and transiently transfected the next day with either hGRwt (**A** and **B**) or hGRdim (**A** and **C**). HepG2 cells that contain endogenous hGR were used in (**D**). Following 24 hours incubation, cells were treated with solvent (DMSO) or 1 µM proteasome inhibitor (MG132) for 1 hour and then, in the absence (−MG132) or presence of MG132 (+MG132), treated with solvent (EtOH) or the compounds Dex, F and CpdA (10^−5^ M) for 16 hours. GRα protein levels were assessed by Western blotting, where GAPDH was probed to ensure equal protein loading. The Western blots shown (**B**–**D** inset) are representative of two to three independent experiments. For quantification (**A**–**D**), the intensity of the GRα and GAPDH bands was determined using UNSCANIT and then the GRα levels were normalized to GAPDH levels and expressed as a percentage (average ± SEM) and plotted. Firstly, the effect of MG132 (+MG132) on unliganded GRα protein levels was investigated (**A**) and compared to GRα levels in the absence of MG132 (−MG132). To compare the effect MG132 treatment on unliganded GRα protein levels one-way ANOVA with a Tukey’s multiple comparisons post-test was conducted on logarithmically transformed data (ns, P > 0.05, **P < 0.01). Thereafter, the effect of MG132 (+MG132) on the extent of hGRwt (**B**), hGRdim (**C**) and endogenous hGRα (**D**) turnover was investigated. The dotted line, on all graphs, represents the fold increase in GRα levels in the presence of solvent (EtOH) and/or absence of MG132 (−MG132) and is set at 1-fold or 100%. To evaluate the effects of ligands on GRα levels in the absence (−MG132) and presence (+MG132) of MG132, statistical analysis was conducted on logarithmically transformed data using a one-way ANOVA with a Dunnett’s multiple comparisons post-test comparing to control (−MG132, EtOH) (ns, P > 0.05; ^#^P < 0.05; ^##^P < 0.001). To analyse the significance of adding MG132 on the extent of ligand-induced GRα protein turnover, a two-way ANOVA was used on logarithmically transformed data followed by a Bonferroni post-test (ns, P > 0.05, **P < 0.01, ***P < 0.001).

To investigate which cellular process, synthesis or degradation, is primarily involved in the turnover of the GRα pool, two inhibitors, cyclohexamide (CHX) and MG132, were used to inhibit translation and proteasomal degradation, respectively. Following treatment with the inhibitors, cells were treated with Dex, F or CpdA, and the effect of translational inhibition by CHX (Supplementary Fig. 2), or proteasomal inhibition by MG132 (Fig. 3) on GRα turnover was determined.

Firstly, inhibiting translation had no significant effect on the extent of ligand-induced GRα protein turnover of hGRwt, hGRdim or the endogenous hGRα (Supplementary Fig. 2). Thus, it was concluded that in the test systems used in this study, GC-mediated regulation of the GRα pool via transcriptional and post-transcriptional processes was negligible.

Secondly, the unliganded hGRwt, but not hGRdim, underwent significant (P < 0.01) basal protein turnover, which was mediated by the proteasome (Fig. 3A). Similarly, the liganded receptor turnover of transiently transfected hGRwt (Fig. 3B) and endogenous hGRα (Fig. 3D) protein by GR dimerizing agents (Dex and F) was abolished by the proteasome inhibitor, MG132, an effect not observed with CpdA-treated hGRwt or endogenous hGRα, or with hGRdim (Fig. 3C). Furthermore, CpdA treatment was not directly inhibiting the function of the proteasome, as demonstrated by effects on the turnover of p53, a protein with a short half-life, in the presence of CpdA (Supplementary Fig. 3), thus confirming that it was CpdA’s ability to abrogate dimerization that was affecting GRα turnover. Taken together, these results provide substantial evidence that the proteasomal degradation pathway plays a predominant role in orchestrating the turnover of the GRα protein and suggests that hGRdim and CpdA-treated hGRwt evade turnover due to their monomeric-favouring GRα conformations.

### Loss of GRα dimerization restricts hyper-phosphorylation at Serine 404

**Figure 4:**
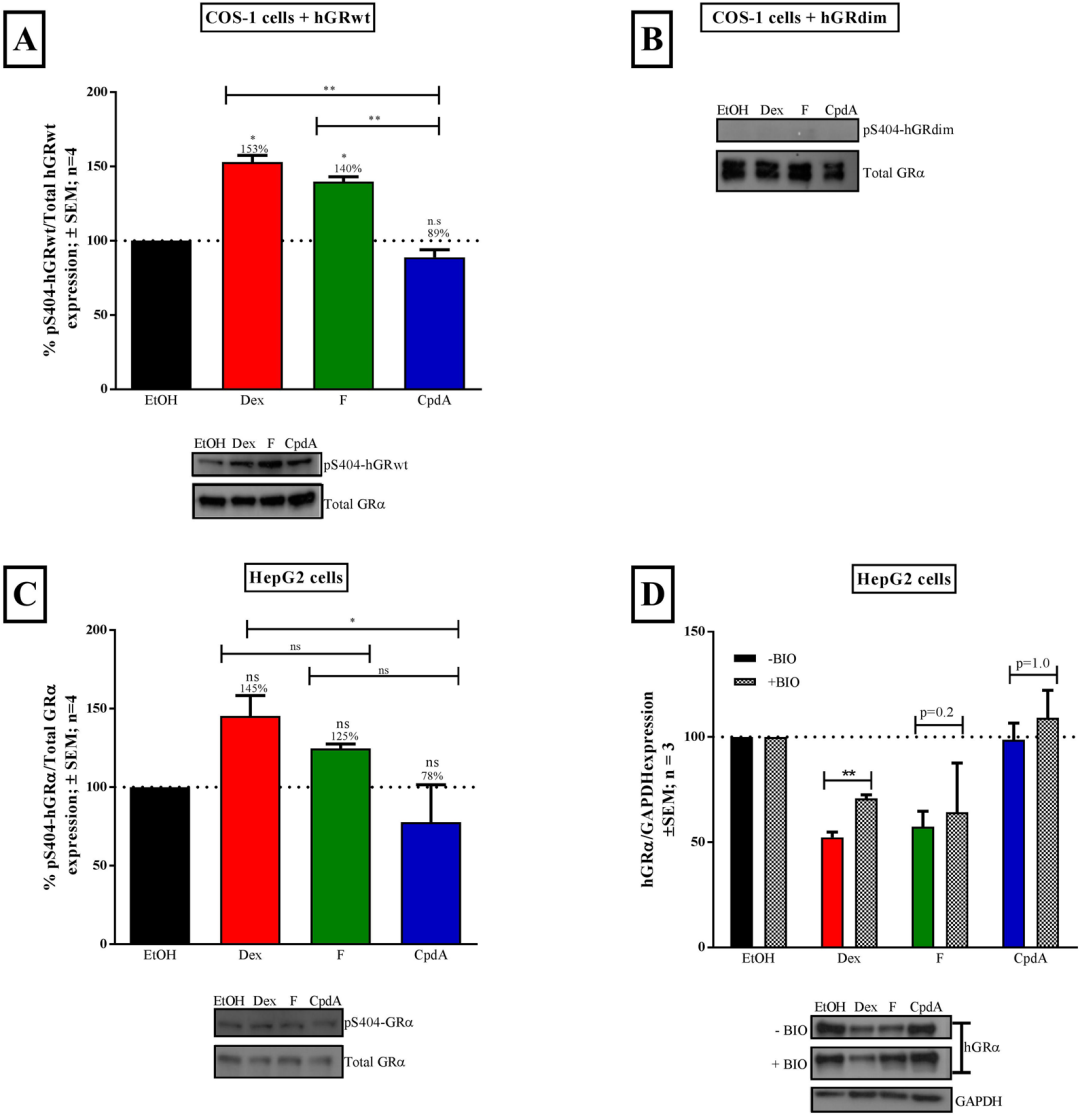
Loss of GRα dimerization, due to CpdA binding or use of hGRdim mutant, restricts hyper-phosphorylation at pS404. COS-1 cells were seeded in a 12 well plate (1 × 10^5^ cell/well) and the next day transiently transfected with either hGRwt (400 ng/well) (**A**) or hGRdim (800 ng/well) (**B**). Following 24 hours incubation, cells were treated with compounds Dex, F and CpdA (10^−5^ M) for 2 hours. This experiment was repeated in HepG2 cells seeded in a 12 well plate (1 × 10^5^ cell/well), containing endogenous GRα (**C**). Additionally, HepG2 cells were treated with 5 μM BIO (GSK3β inhibitor) for 1 hour prior to treatment with compounds (**D**). pS404-GR levels in (**A**–**C**) were detected using Western blotting, which were quantified using UNSCANIT. Blots were stripped and re-probed for total GRα protein content for normalization. In the case of (**D**), total hGRα was detected and normalized to loading control GAPDH. Normalized values were then plotted and expressed as a percentage relative to solvent (EtOH), which was set at 100% and is represented by the dotted line. A representative blot from a single experiment is shown. For statistical analysis, of hGRwt (**A**) and endogenous hGRα (**C**), a one-way ANOVA followed by a Tukey’s multiple comparisons post-test (ns, P > 0.05, *P < 0.05, **P < 0.01 and ***P < 0.001) was conducted on logarithmically transformed data. An unpaired two-tailed t-test with Welch’s correction was conducted comparing hGRα protein levels in the absence or presence of BIO, following Dex, F or CpdA treatment in (**D**). Full-length blots are presented in Supplementary Fig. S7.

Hyper-phosphorylation of GRα at S404 in humans, mediated by glycogen synthase kinase 3β (GSK3β), following treatment with dimerization promoting Dex, has been shown to decrease overall GRα stability^37^. This led to us to postulate that a loss of GRα dimerization may restrict phosphorylation of GRα at S404, thus preventing receptor turnover and maintaining GRα stability. To test this hypothesis, COS-1 cells, transiently transfected with hGRwt or hGRdim, or HepG2 cells, containing endogenous GRα, were treated with solvent, Dex, F or CpdA. Phospho-S404 GRα levels were then determined by Western blotting (Fig. 4).

Dimerization promoting GRα ligands, Dex and F, resulted in a significant (P < 0.05) increase in transiently transfected hGRwt phosphorylation at S404 (Fig. 4A), also observed with endogenous hGRα, in the HepG2 cells (Fig. 4C). In stark contrast, loss of GRα dimerization through the use of the dimerization deficient mutant, hGRdim, did not result in any hyper-phosphorylation, in fact, phosphorylation seemed to be completely absent and even basal phosphorylation was undetectable (Fig. 4B), unlike with the hGRwt (Fig. 4A). In support of the hGRdim result, CpdA treatment substantially reduced phosphorylation of hGRwt (Fig. 4A) and endogenous hGRα (Fig. 4C). Furthermore, in the HepG2 cells, inhibition of the Dex mediated hyper-phosphorylation at S404, using the GSK3β inhibitor, BIO (Supplementary Fig. 4), resulted in significant (P < 0.01) restoration of GRα protein levels (Fig. 4D).

To summarize, this novel link between GRα conformation (i.e. monomer versus dimer) and hyper/hypo-phosphorylation at S404, confirms that the dimerization state of the GRα influences the post-translational processing of the GRα and explains, in part, one of the ways in which ligand-induced turnover of predominantly monomeric GRα is prevented.

### Loss of GRα dimerization restricts interaction of GRα with FBXW7α

To further explore the link between GRα conformation, phosphorylation at Ser404 and GRα turnover, we speculated that the E3 ligase, FBXW7α, may be involved as the GRα has recently been identified as a novel client of this E3 ligase, whose interaction with the receptor^44^ is dependent on prior GC-mediated hyper-phosphorylation of GRα at Ser404, by GSK3β^37^.

**Figure 5:**
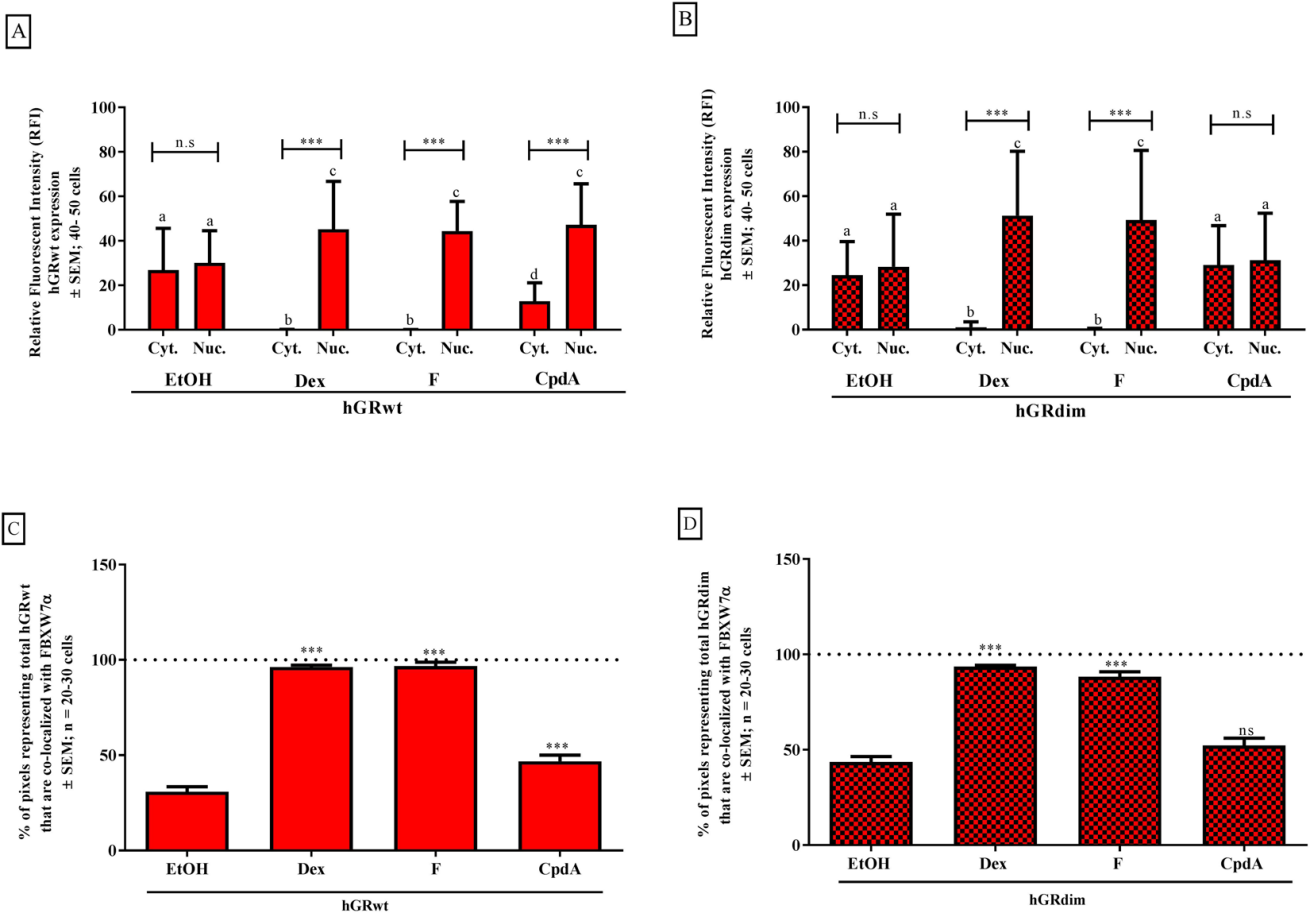
Ligand-dependent subcellular localization of GRα modulates its co-localization with endogenous FBXW7α. COS-1 cells were seeded into a 10 cm dish (1 × 10^6^ cells) and transiently transfected with either hGRwt (**A** and **C**) or hGRdim (**B** and **D**). Following 24 hours incubation, cells were re-plated and treated with solvent (EtOH), Dex, F or CpdA (10^−5^ M) for 3 hours. Thereafter, cells were fixed, permeabilised, and immunofluorescence conducted, with antibodies specific for GRα and FBXW7α. Cells were then imaged using a confocal microscope. For the quantification of the subcellular localisation of (**A**) hGRwt or (**B**) hGRdim the relative fluorescence intensity (RFI) of the red (GRα) pixels was calculated for individual cells by selecting regions of interest (ROI), and plotted. In addition, the co-localisation of GRα and FBXW7α, in terms of hGRwt (**C**) or hGRdim (**D**), was determined using the weighted co-localisation coefficients, and expressed as a percentage, where the horizontal dotted line represents 100% co-localization of GRα (hGRwt or hGRdim) with FBXW7α. To compare the cytoplasmic and nuclear expression of hGRwt (**A**) or hGRdim (**B**) within a treatment group statistical analysis was conducted on logarithmically transformed data using a two-way ANOVA followed by a Bonferroni multiple comparisons post-test (ns, P > 0.05 and ***P < 0.001). In addition, comparing localization of the hGRwt (**A**) or hGRdim (**B**) of all samples to each other, a one-way ANOVA with a Tukey’s multiple comparisons post-test was conducted on logarithmically transformed data (for a, b, c and d, letters that are the same represent no significant difference between values whilst letters, which are different are significantly different from each other P < 0.05). Comparing ligand-induced co-localization of hGRwt (**C**) or hGRdim (**D**) with FBXW7α statistical analysis was conducted on logarithmically transformed data using a one-way ANOVA with a Dunnett’s multiple comparisons post-test, comparing experimental values to the solvent (EtOH) (ns, P > 0.05 and ***P < 0.001).

As FBXW7α is predominantly localized to the nucleoplasm^62^, nuclear localization of the GRα would be a prerequisite for an interaction between these two proteins. Thus to determine the effect of the dimerization state of GRα on the subcellular localization and co-localization with FBXW7α we conduced indirect immunofluorescence (Fig. 5). Specifically, COS-1 cells transfected with either hGRwt or hGRdim, were treated with solvent, Dex, F or CpdA for 3 hours and immunofluorescence was performed using antibodies specific to GRα and FBXW7α (Supplementary Fig. 5A and B). FBXW7α was mostly nuclear with minimal expression detected in the cytoplasm across all treatment conditions (Supplementary Fig. 5C and D). The unliganded GRα receptor, whether hGRwt or hGRdim, was evenly distributed throughout the cytoplasm and the nucleus, while, treatment with the dimerization promoting GCs, Dex and F, resulted in total nuclear translocation of hGRwt and hGRdim (Fig. 5A and B; Supplementary Fig. 5A and B). Similarly, treatment with the dimerization abrogating GC, CpdA, resulted in significant (P < 0.001) translocation of hGRwt, however, unlike with Dex and F treatment, a substantial amount of hGRwt still resided in the cytoplasm (Fig. 5A; Supplementary Fig. 5A). In contrast, CpdA did not induce translocation of hGRdim (Fig. 5B; Supplementary Fig. 5B). Co-localization of the GRα with FBXW7α indicates that only 30% to 44% of the unliganded hGRwt and hGRdim, respectively, co-localizes with FBXW7α (Fig. 5C and D), which may be due to the fact that a large portion of the hGR does not occupy the same space as FBXW7α. This argument also holds true for the treatment conditions with the Dex, F and CpdA. Specifically, Dex and F, which resulted in a significant (P < 0.001) increase in the co-localization of hGRwt and hGRdim with FBXW7α, with almost all (just less than 100%) of hGR occupying the same space as FBXW7α (Fig. 5C and D), also induced significant (P < 0.001) and almost total nuclear translocation of both the hGRwt and hGRdim (Fig. 5A and B). Furthermore, CpdA also resulted in a significant (P < 0.001), but lower increase (47%) in the amount of hGRwt co-localized with FBXW7α (Fig. 5C) that correlates with the significant (P < 0.001), but diminished, ability of CpdA to induce nuclear translocation of hGRwt (Fig. 5A). In contrast CpdA, did not significantly affect the co-localization of FBXW7α with hGRdim (Fig. 5D), which reflects the inability of CpdA treatment to induce nuclear translocation of hGRdim (Fig. 5B). Taken together, these results suggest that co-localization of GRα and FBXW7α does not provide the link between GRα conformation, phosphorylation at Ser404 and GRα turnover but rather correlates with the extent of nuclear localization of GRα.

**Figure 6:**
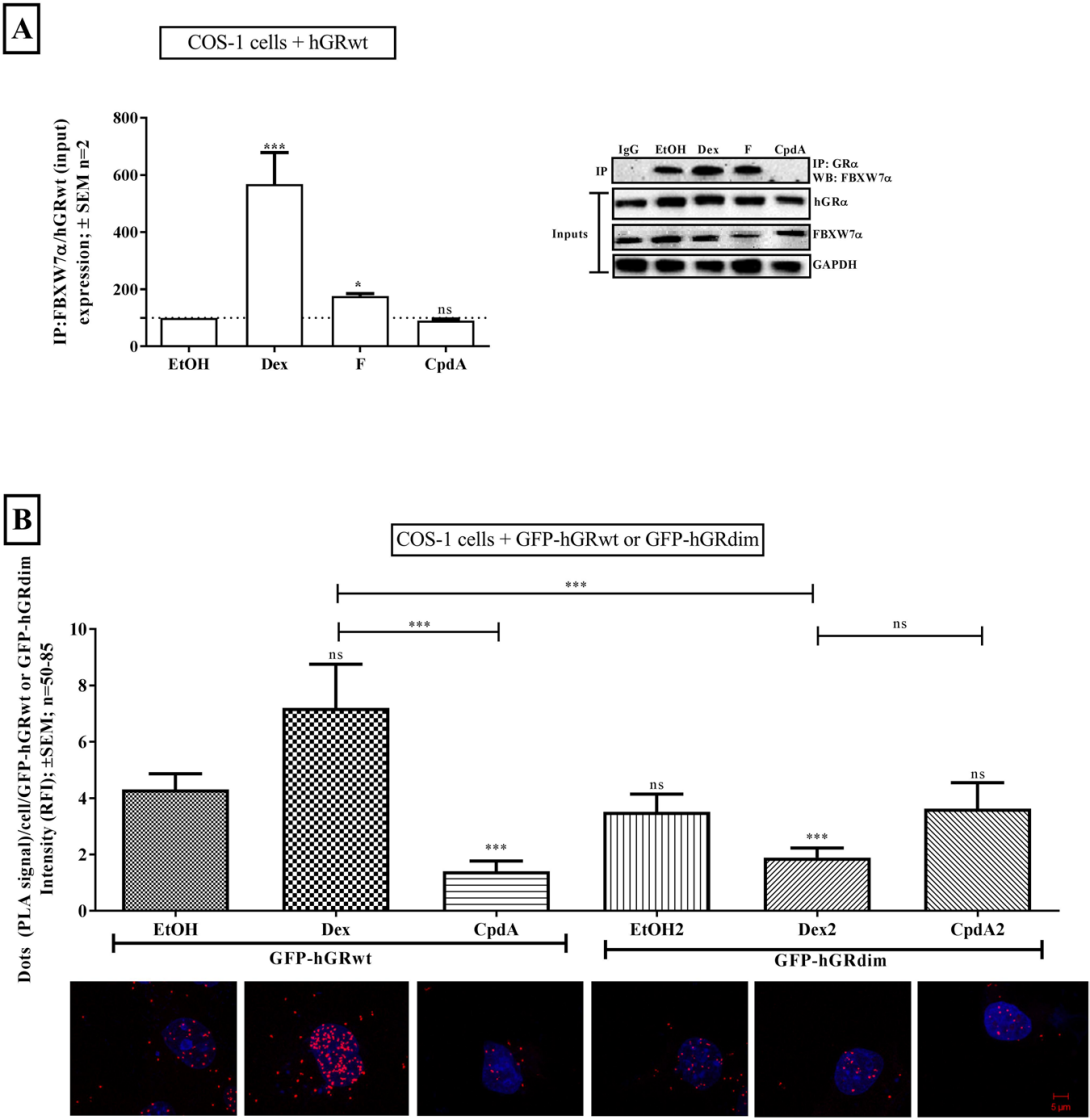
Loss of GRα dimerization modulates its interaction with FBXW7α. COS-1 cells were seeded, transfected and re-plated into 8 well chambers. Following 24 hours incubation, cells were treated with solvent (EtOH), Dex, F or CpdA (10^−5^ M) for 3 hours. For the co-IP (**A**) experiment, cells were lysed after compound treatment and FBXW7α was immuno-precipitated with a GRα antibody. Western blotting was conducted on Inputs to probe for GRα, FBXW7α and GAPDH and on IP to probe for FBXW7α. A representative blot of three independent experiments is shown (IP: row 1 and Inputs: rows 2–4). For quantification, the intensity of, hGRwt, FBXW7α and GAPDH were determined using the MyECL Image Software Analysis. Moreover, the hGRwt/FBXW7α interaction was normalized to hGRwt input expression and expressed as a percentage relative to EtOH. The dotted line on the graph represents the hGRwt/FBXW7α interaction in the presence of solvent (EtOH), and is set at 100%. For the PLA (**B**), following treatment cells were fixed, permeabilized and PLA conducted using specific antibodies for GRα and FBXW7α, after which cells were imaged. A representative image of individual cells from the GFP-tagged GRα and FBXW7α (B, inset below graph) experiment, is shown. In these representative images the PLA signal is observed as distinct red spots and the cell’s nucleus is depicted by the blue DAPI stain. For quantification of the GFP-tagged GRα and FBXW7α interaction (**B**), the PLA signal (dots/cell) was quantified using the IMAGEJ Software and normalized to the GRα concentration (i.e. GFP-signal (RFI)), which was determined using the ZENN 2012 Software Analysis, and plotted. To compare GRα hGRwt/FBXW7α interaction in response to the test compounds relative to solvent (EtOH) using Co-IP (**A**), statistical analysis was conducted on logarithmically transformed data using a one-way ANOVA followed by a Dunnett’s multiple comparisons post-test (ns, P > 0.05, *P < 0.05 and ***P < 0.001). PLA analysis of GFP-hGRwt or GFP-hGRdim interacting with FBXW7α in response to the test compounds (**B**) were compared using one-way ANOVA followed by a Tukey’s multiple comparisons post-test on logarithmically transformed data (ns, P > 0.05 and ***P < 0.001). Full-length blots are presented in Supplementary Fig. S8.

Thus co-immunoprecipitation analysis was performed in COS-1 cells transfected with hGRwt to determine whether loss of receptor dimerization would influence the ability of FBXWα to interact with GRα. The dimerization abrogating GC, CpdA, indeed did not induce an interaction of the receptor with FBXW7α, in stark contrast to the potent, synthetic dimerization promoting GC, Dex, which led to a significant (P < 0.001) increase in the association of hGRwt with the E3 ligase, FBXW7α (Fig. 6A). Furthermore, following treatment with F a substantial and significant (P < 0.05) almost two-fold increase was seen (Fig. 6A).

To validate the co-immunoprecipitation results a proximity ligation assay (PLA) was conducted using COS-1 cells transiently transfected with GFP-hGRwt or GFP-hGRdim (Fig. 6B). In support of the co-immunoprecipitation result, promoting dimerization of GFP-hGRwt through treatment with Dex, resulted in a substantial increase in the association of the wild-type receptor with FBXW7α (Fig. 6B). Moreover, loss of dimerization of GFP-hGRwt, following CpdA treatment, significantly (P < 0.001) restricted the interaction of the receptor with the E3 ligase (Fig. 6B), as in Fig. 6A. Interestingly, no significant (P > 0.05) ligand-induced increases in the interaction of the dimerization deficient GRα mutant, GFP-hGRdim, with FBXW7α, were observed relative to the unliganded GFP-hGRwt (Fig. 6B). In addition, the ability of dimerization promoting Dex to induce an interaction between GRα and FBXW7α was significantly (P < 0.001) restricted with the use of GFP-hGRdim, relative to GFP-hGRwt (Fig. 6B), which cannot be ascribed to a lack of Dex-induced co-localization between hGRdim and FBXW7α as shown in Fig. 5D.

In summary, loss of GRα dimerization, either by CpdA treatment or the use of the dimerization deficient hGRdim, reduces the interaction of the receptor with FBXW7α. Together these results from the co-IP and PLA solidify a role for GRα dimerization in promoting an interaction with the E3 ligase, FBXW7α, suggesting that it provides the link between GRα conformation, phosphorylation at Ser404 and GRα turnover.

### Co-treatment with CpdA lessens the extent of Dex-induced GRα protein turnover, thereby partially restoring GRα levels

It is clear that the conformation of the GRα, specifically its dimerization state, plays a vital role in ligand-induced receptor turnover, with dimerization inducing ligands like Dex inducing turnover, while dimerization abrogating ligands like CpdA restricting turnover. This led us to the hypothesis that co-treatment of Dex with CpdA may lessen the effect of Dex on hGRwt turnover.

**Figure 7:**
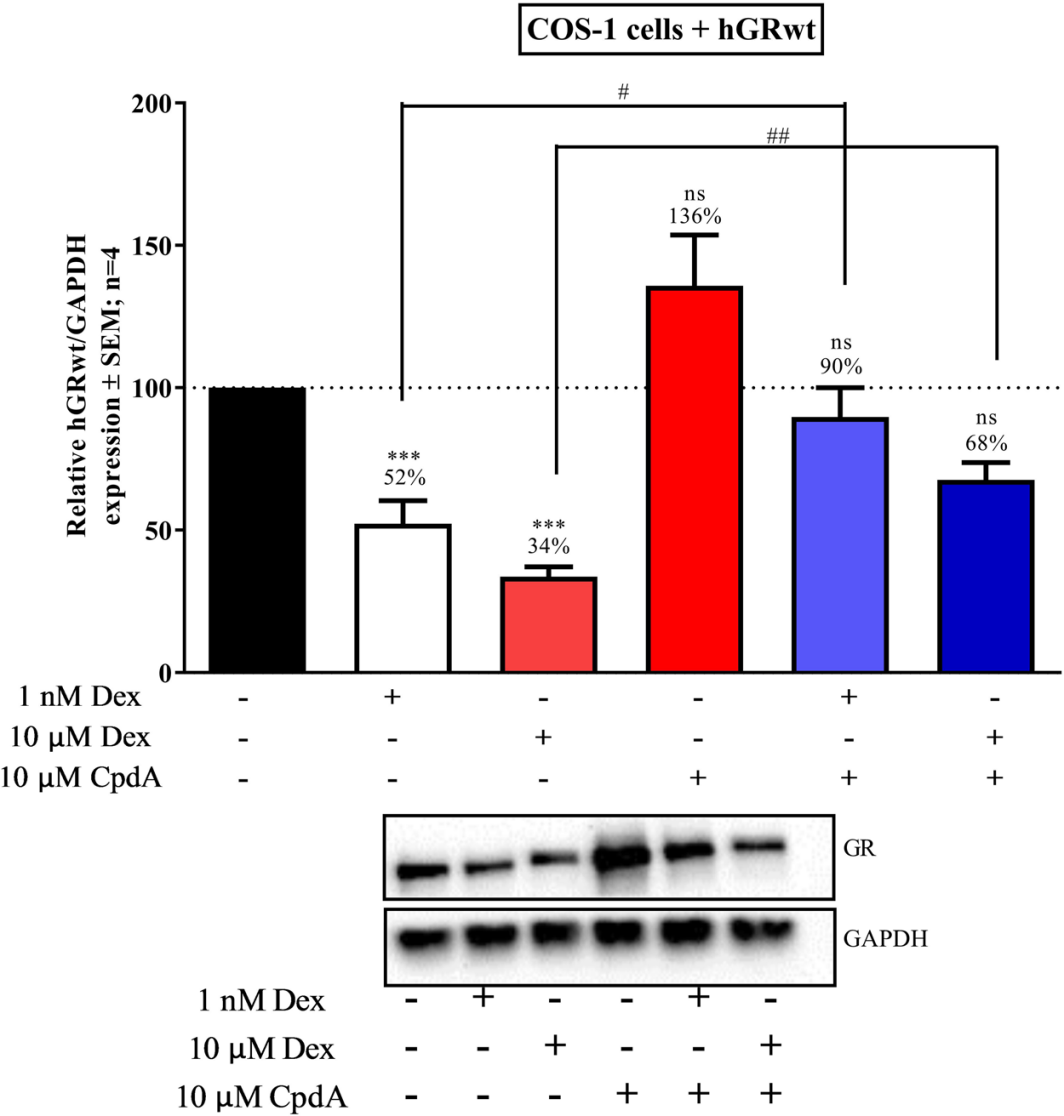
Co-treatment with CpdA partially restores hGRwt protein levels. COS-1 cells were seeded into a 24 well plate (5 × 10^4^ cells/well) and transiently transfected with hGRwt. Following 24 hours incubation, cells were treated individually with solvent (EtOH), Dex (1 μM or 10 μM) or CpdA (10 μM) or with CpdA (10 μM) in combination with Dex (1 μM or 10 μM) for 24 hours. hGRwt protein expression was confirmed by Western blotting where GAPDH was probed to ensure equal protein loading. Western blot shown (inset) is representative of four independent experiments. For quantification, the intensity of the hGRwt and GAPDH bands were determined using My ECL Image Analysis software and hGRwt levels were then normalized to GAPDH expression. hGRwt expression is expressed as a percentage (average ± SEM) of hGRwt expression in presence of the solvent (EtOH), which was set at 100% (dotted line). To determine the effect of each treatment condition on hGRwt protein expression relative to the solvent (EtOH), statistical analysis was conducted on logarithmically transformed data using a one-way ANOVA with a Dunnett’s multiple comparisons post-test (ns, P > 0.05 and ***P < 0.001). To assess the ability of CpdA to preserve hGRwt expression in the presence of Dex an unpaired two-tailed t-test with Welch’s correction was conducted comparing hGRwt expression following Dex treatment (either 1 μM or 10 μM) alone to Dex treatment in combination with CpdA (10 μM) (^#^P < 0.05, ^##^P < 0.01). Full-length blots are presented in Supplementary Fig. S9.

Consistent with previous results hGRwt protein levels are significantly (P < 0.001) reduced by Dex, but not by CpdA treatment (Fig. 7). Co-treatment of Dex with CpdA, however, significantly (P < 0.05) diminishes the extent of Dex-induced GRα protein down-turnover. Thus CpdA demonstrates an ability to restore hGRwt protein levels in the presence of a dimerization inducing ligand like Dex.

## Discussion

Side effects and resistance to treatment remain a major concern in the therapeutic use of glucocorticoids, such as Dex. GC resistance is characterized by a loss of GC sensitivity, which has been shown to be directly proportional to the available functional pool of GRα^14–16,63^. The functional pool of GRα may be affected by various factors, including the well documented ligand-induced down-regulation of the receptor^27,31,36,37,50,57^. The factors that influence the extent of ligand-induced receptor turnover are, however, not entirely understood. In the current study we present evidence for a novel role for the dimerization state of the GRα in mediating GC-mediated GRα turnover.

GRα dimerization is considered an essential step in GC-mediated transactivation and occurs via two dimerization interfaces, one in the DNA-binding domain (DBD) and the other in the ligand binding domain (LBD) of the receptor^3^. Loss of GRα dimerization has been engineered by point mutations at these sites, for example the hGRdim mutation (A458T) occurs in the DBD, while the hGRmon^55^ (A458T; I628A) is mutated at an additional site in the LBD. The GRdim mutant has been widely utilised in studies exploring the role of GRα dimerization, however, of late the loss of dimerization via the GRdim has been controversial, with some studies^55,64^ concluding that the GRdim demonstrates an ability to dimerize, while others^52,54^ suggest that GRdim is less efficient at promoting dimerization than the GRwt. A recent paper^54^ very nicely shows in living cells, that the equilibrium dissociation constant (Kd) for dimerization in the presence of Dex is substantially lower for GRwt (3.00 µM) than for GRdim (6.11 µM), which is closer to the Kd of the unliganded GRwt receptor (7.40 µM), suggesting that the GRdim mutation impairs dimerization resulting in a loss, rather than an abrogation, of dimerization. In contrast, CpdA, a non-steroidal ligand of the GR initially identified as a SGRM^65^, has been shown to not only result in a loss of receptor dimerization but to totally abrogate GRwt dimerization^52,53,55^. The idea to investigate the role of ligand-induced dimerization in mediating GR turnover thus originated not only from the fact that CpdA abrogates dimerization, but also from the observation that CpdA treatment does not lead to receptor downregulation^36,49,51^. Since both GRdim and CpdA result in a loss of GR dimerization, in the current study their effects were contrasted to that of the endogenous GR ligand, F, and the therapeutic glucocorticoid, Dex, both of which elicit dimerization of the GRwt^55^.

The dimerization promoting GC’s, Dex and F, were shown to result in up to 80% reductions in the hGRwt pool after 72 hrs, as well as a significantly accelerated rate of receptor turnover and a reduction in the receptor half-life. The absolute values for the half-lives reported in the current study where considerably higher than previously reported values^36,37,43,47,57,66^ however, if the fold reductions are compared, these values are comparable to the literature. In contrast, the current study confirmed^36,49,51,65,67,68^ not only that CpdA treatment does not lead to receptor turnover, but that both receptor turnover rate and receptor half-life is substantially different from that of unliganded receptor, which is likely due to the fact that CpdA had been shown to abrogate ligand-independent dimers^52^. Furthermore, loss of dimerization, through the use of hGRdim, also prevented the dimerization promoting ligands (Dex and F) from inducing receptor turnover, thus reinforcing the link between GRα dimerization and ligand-induced GRα protein turnover.

Although the GR turnover rate is governed by two opposing processes, synthesis and degradation^3^, the current study identified minimal regulation at the level of synthesis, but strongly implicated the proteasome in orchestrating ligand-induced reductions in the GRα pool, in accordance with previous studies^30,31^. Specifically, the current study showed that dimerized hGRwt turnover was significantly inhibited by the proteasome inhibitor, MG132. Although a slight increase in unliganded hGRdim protein expression was noted in the presence of MG132, this increase was substantially less than that observed for unliganded hGRwt and suggests that although basal turnover of hGRdim via the proteasome occurs, it is not as drastic as for unliganded hGRwt protein turnover and is likely due to hGRdim’s impaired ability to form GRα dimers^52,54^. In addition, the possibility that CpdA had a direct inhibitory effect on the function of the proteasome was ruled out by investigating, the effect of CpdA treatment on p53 turnover.

For the UPS to recognize substrates for degradation PTMs, such as phosphorylation, are required. Galliher-Beckley *et al*.^37^ demonstrated that dimerization promoting GCs induce GSK3β mediated hyper-phosphorylation of GRwt at S404 thus increasing receptor turnover, which is supported by data from the current study demonstrating hyper-phosphorylation at Ser404 with the dimerization promoting GCs, F and Dex. Additionally, we demonstrate that that restricting hyper-phosphorylation at S404 through the use of a GSK inhibitor (BIO) partially prevents the GRwt turnover elicited by dimerization promoting GCs (i.e. Dex). In contrast and unlike the dimerization promoting GCs, dimerization abrogating CpdA substantially restricted phosphorylation of the receptor at S404, while phosphorylation of the dimerization deficient mutant, hGRdim, was in fact undetectable across all treatment conditions. Thus, lack of ligand-induced hyper-phosphorylation of GRα at S404 is identified as one of the molecular mechanisms, which contribute to the stability of monomeric GRα.

The E3 ligase, FBXW7α, recognizes hyper-phosphorylation of GRα at S404 and regulates the receptor turnover in a ligand-dependent manner^44^. In order for FBWX7α to mediate successful ligand-induced GRα turnover, a physical interaction between the E3 ligase and the receptor is required^44^. Thus, as FBXW7α is a nuclear protein^62^, this implies that the interaction with GR requires nuclear localization of the receptor. We show that while F and Dex can successfully translocate both the GRwt and the GRdim to the nucleus resulting in almost 100% co-localization with FBXW7α, CpdA, in contrast, only promotes slightly increased nuclear localization of the GRwt, which has also been observed previously in stromal myofibroblasts^69^ and COS-1 cells^52,70^. In addition the co-localization of CpdA-induced GRwt with FBXW7α is substantially lower than that induced by F and Dex. CpdA, however, did not increase nuclear localization of the GRdim in the current study, in contrast to previous studies that had shown nuclear localization by CpdA-liganded GRdim, albeit with a decrease in maximal import levels^52,70^. Furthermore, co-immunoprecipitation and PLA assays report a physical interaction between FBXW7α and Dex-induced GRwt, but not GRdim, despite the fact that co-localization studies show that Dex can induce co-localization of both receptors with FBXW7α. CpdA binding to the GRwt and GRdim, on the other hand, does not induce a physical interaction between FBXW7α via either receptor. Thus the physical interaction between GR and FBXW7α appears to be mainly influenced by the phosphorylation of the receptor at S404. Interestingly, apart from the role of FBXW7α in mediating GRα turnover and thus diminishing the anti-inflammatory actions of GCs, FBXW7α has been implicated in both the attenuation of inflammation via degradation of C/EBPδ^71^ and the potentiation of inflammation through degradation of p100, an inhibitor of non-canonical NF-κB signalling^72^.

Although the current study generated loss of dimerization by using either the dimerization deficient mutant, GRdim, or the monomerization biased ligand, CpdA, our results show that the two scenarios do not always produce exactly the same results. Specifically, although both scenarios result in a decrease in GR turnover and reduced interaction with FBXW7α, CpdA only reduces basal S404 phosphorylation while GRdim shows no phosphorylation at this site. CpdA has previously been shown to also reduce GR phosphorylation at Ser211 and S226^36,65,73^, which correlates with transactivation efficacy and potency, but to our knowledge the GRdim has not been investigated for effects on phosphorylation at these two sites, nor at S404. Furthermore, while GRdim can be translocated to the nucleus by dimerization promoting ligands, CpdA can partially translocate only GRwt, but not GRdim, in the current study. With CpdA abrogating even basal dimerization, while the GRdim being deficient in dimerization, only results in a loss rather than abrogation of receptor dimerization, it may be tempting to ascribe the difference between the two scenarios to the difference in the extent of loss of dimerization. However, while investigation of the double dimerization mutant, GRmon^55^, may shed light on the issue, it is important to not discount the fact that the two scenarios may also result in different conformations of the GR that extends beyond that of loss of dimerization and which produce further mechanistic differences^67,74,75^. For example, the GRdim mutation may result in a GR conformation that is not available for phosphorylation at S404.

Combinatorial strategies are important new approaches to improve the therapeutic index of drugs and as such CpdA was shown to restore Dex-induced downregulated hGRwt protein levels in the current study. CpdA has previously been combined with GCs, primarily in studies investigating the amelioration of side-effects in an inflammatory setting, where the combinatorial approach has generally led to an improved therapeutic profile^65,74,76–78^. However, to our knowledge only one study has investigated the combinatorial effects of CpdA and Dex on GR levels in myofibroblasts^69^ and in contrast to the findings of the current study, demonstrated that co-treatment augmented, rather than rescuing, GR protein downregulation. Interestingly CpdA in combination with the proteasome inhibitor, Bortezomib (BZ), has been investigated in the context of lymphoma and multiple myeloma cells and shows that BZ enhances CpdA’s dissociated properties and that CpdA on its own prevents down-regulation not only of the GR, but also of FKBP51, a GR chaperone protein upregulated by Dex that is involved in acquired GC resistance^67^.

**Figure 8:**
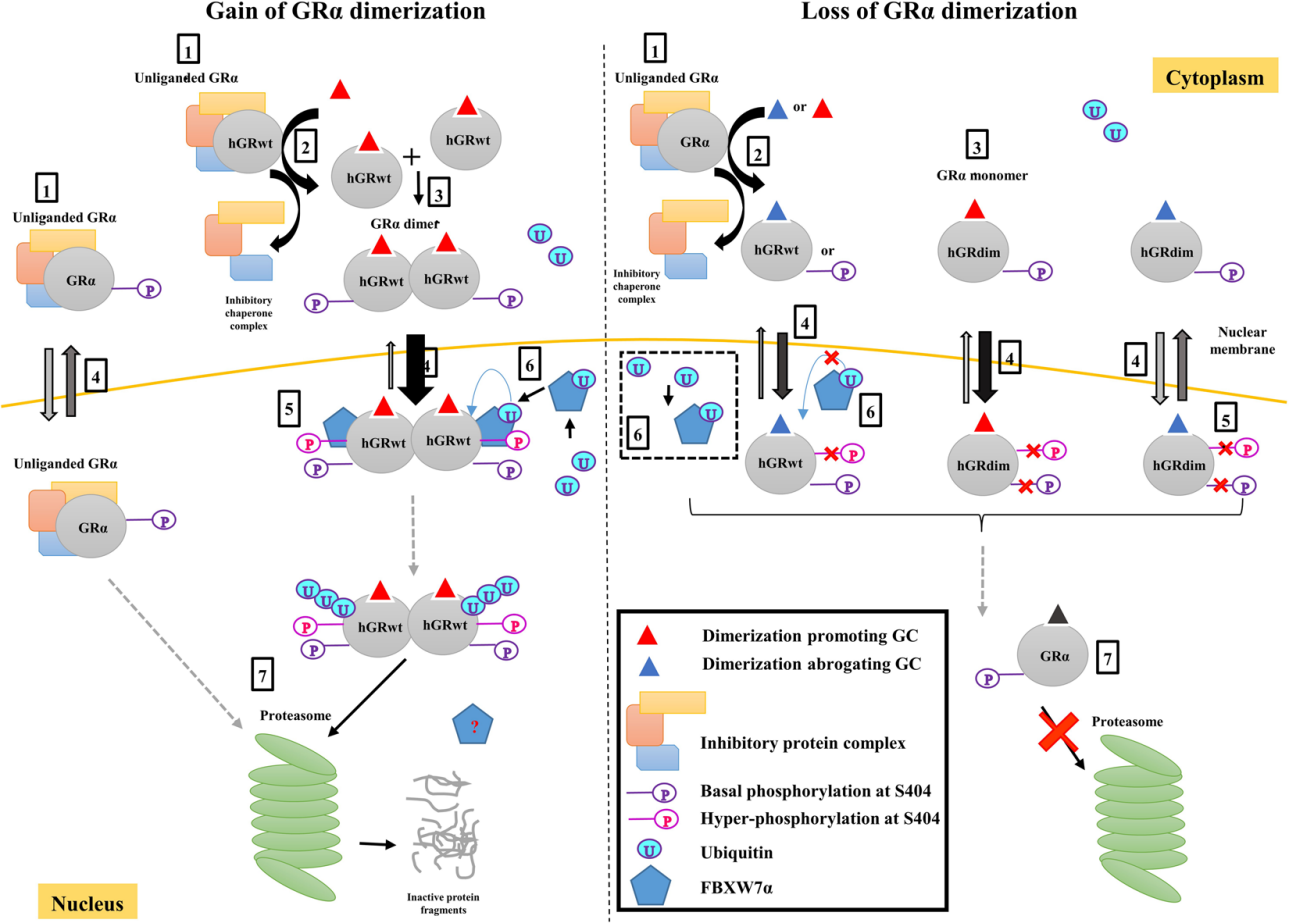
Proposed model comparing the effects of gain and loss of GRα dimerization in GRα turnover (see solid box on model, for definitions). 1. Unliganded, hypo-phosphorylated GRα is primarily cytoplasmic bound to an inhibitory chaperone complex. 2. Ligand binding causes dissociation of the inhibitor chaperone complex 3. Upon ligand binding of a dimerization promoting GC (i.e. Dex or F) the hGRwt dimerizes. However, in the case of binding of the dimerization abrogating compound, CpdA, to the hGRwt, the receptor does not dimerize. Moreover ligand-binding to the hGRdim also does not result in receptor dimerization. 4. The liganded-GRα translocates to the nucleus. The unliganded GRα, is evenly distributed between cytoplasm and nucleus. The thickness of the arrows refers to extent of movement. 5. Once in the nucleus, the dimerized hGRwt undergoes hyper-phosphorylation at Ser404. In contrast, loss of GRα dimerization restricts hyper-phosphorylation at this site. 6. In the case of gain of hGRwt dimerization, phosphorylation at S404 facilitates binding of the E3 ligase, FBXW7α, which binds ubiquitin and mediates hGRwt ubiquitination. Unlike gain of GRα dimerization, loss of GRα dimerization restricts, albeit partially, the binding of the E3 ligase, FBXW7α. 7. The GRα is then eventually targeted for degradation by the proteasome, in the case of dimeric GRα, but not in the case of monomeric GRα. The dotted arrow in the model indicates that cellular events, not shown, may occur.

Collectively the results from the current study propose a model (Fig. 8) whereby efficient degradation of the GR protein pool relies on a dimeric conformation of the receptor. Essentially the dimerization state of the receptor influences two molecular mechanisms, namely hyper-phosphorylation at S404 and subsequent interaction of the GR with the E3 ligase, FBXW7α, ultimately affecting its turnover.

In conclusion, with acquired resistance to GC treatment gaining traction and in many cases posing major clinical challenges in treating chronic inflammation, the elucidation, by the current study, of the role of receptor dimerization in receptor turnover provides novel molecular insights, which may be exploited in an attempt to reverse or counteract resistance. Thus abrogation of GR dimerization may be a worthwhile molecular target for the rational design of conformationally biased ligands of the GR that improves the therapeutic profile not only through a decrease in side-effects, but also by reducing the likelihood of GC resistance due to increased GR turnover.

## Methods

### Reagents and Test Compounds

Dexamethasone (Dex) and cortisol (F) were purchased from Sigma-Aldrich. Compound A (CpdA; 2-(4-acetoxyphenyl)-2-chloro-*N*-ethylethylammonium chloride) was synthesized as previously described^79^. All stock solutions were prepared in ethanol to a final concentration of 1 M and stored at −20 °C. For treatments, stock solutions were diluted to final concentrations of 10^−5^ M for Dex, F and CpdA or 10^−6^ M for Dex so that the final concentration of EtOH did not surpass 0.1% (v/v). The proteasome inhibitor, MG132, translational inhibitor cycloheximide (CHX), and 6-bromoindirubin-3′-oxime (BIO) a GSK3α/β inhibitor was purchased from Sigma-Aldrich and prepared as per the manufactures instructions.

### Cell culture and transfection

African green monkey kidney **(**COS-1) cells and the human liver carcinoma cell line (HepG2), containing endogenous GRα, were purchased from American Type Culture Collection (USA). Cells were maintained in high glucose (4.5 g/ml) Dulbecco’s modified Eagle’s medium (DMEM) supplemented with 10% foetal calf serum (FCS), 1.5 g/L sodium bicarbonate, 0.11 g/L sodium pyruvate and 100 IU/ml penicillin and 100 µg/ml streptomycin (1% Pen/Strep). For the HepG2 cells, additional L-glutamine was added to a final concentration of 2 mM. The cell lines were maintained at a temperature of 37 °C, 90% humidity and 5% CO_2_ in T75 tissue culture flasks. COS-1 cells 24 hours after seeding were transiently transfected with pRS-hGRα (hGRwt)^80^, pHisGRA458T (hGRdim)^61^, pEGFP-C2-GR (GFP-hGRwt)^81^ or pEGFP-C2-GRA477T (GFP-hGRdim)^81^ using XtremeGENE HP Fugene transfection reagents as described by manufacturer.

### Western blotting

Cells were plated and induced in serum free DMEM with the test panel at concentrations and for times as indicated in Figure legends. After washing with phosphate buffered saline (PBS), cells were harvested in SDS sample buffer (100 mM Tris-HCl pH 6.8, 5% (w/v) SDS, 20% (v/v) glycerol, 2% (v/v) β-mercaptoethanol and 0.1% (w/v) bromophenol blue) after which, standard Western blotting procedures were followed using anti-GRα (sc-8992, Santa Cruz), anti-GAPDH (sc-47724, Santa Cruz), anti-pS404-GR^37^ (Gift from J.Cidlowski, National Institute of Environmental Health Sciences, USA), anti-p53 (#2524d, Cell Signalling) and anti-FBXW7α (ab109617b, Abcam). Equal loading was ensured by the use of a loading control (GAPDH). Bands were visualized using chemiluminesence and the MyECL Imager (Thermo Scientific, USA). For data presentation see Figure legends.

### Whole cell GRα-binding

COS-1cells, were seeded, transfected (hGRwt and hGRdim) and treated in serum free DMEM with the test panel at concentrations and for times as indicated in Figure legends. Following each treatment, cells were washed with pre-warmed 0.2% BSA-PBS and a whole cell binding experiment performed as previously described^82^. Briefly, unsupplemented DMEM containing 20 nM [^3^H]-Dex (with a specific activity of 68 Ci/mmol; obtained from AEC Amersham) in the absence (total binding) or presence (non-specific binding) of 10 μM unlabeled Dex, was added to wells (500 μl/well) and incubated at 37 °C for 4 hours. After incubation cells were washed with ice-cold 0.2% BSA-PBS, lysed and counts per minute (cpm) determined using a scintillation counter. Total protein concentration was determined using the Bradford method^83^, which was used to normalize results (cpm/mg protein) and specific binding was then calculated. For data presentation see Figure legends. All experiments were evaluated for ligand depletion and counting efficiency (CE), which was less than 10% and approximately 43%, respectively.

### Co-immunoprecipitation (Co-IP)

Following, seeding, transfection and treatment with the test panel as indicated in Figure legends, COS-1 cells were lysed in RIPA Buffer (#R2078, Sigma Aldrich) supplemented with a Complete Mini Protease Inhibitor Tablet (Roche). Following a freeze and thaw cycle, cells were harvested and the supernatant collected (inputs - set aside). Protein A/G PLUS-Agarose beads (#sc-2003, Santa Cruz) were pre-blocked for 1 hour at 4 °C with salmon sperm DNA (11 mg/ml stock) (Thermos Scientific) in IP dilution buffer (0.01% SDS, 20 mM Tris pH 8, 1.1% Triton-X-100, 167 mM NaCl, 1.2 mM EDTA, 1 x protease inhibitor tablet). Following pre-blocking of the beads, the sample supernatant was pre-cleaned, to minimize nonspecific binding to the beads, with 15 μl of 50% slurry of beads in IP dilution buffer while rotating for 1 hour. Once pre-cleaned, samples were centrifuged at 5500 × g for 1 minute and the supernatant collected to which the anti-GRα antibody (sc-8992), was added and rotated overnight at 4 °C. After incubation the pellet was washed 6-times with wash buffer (0.1% SDS, 1% Triton x100, 2 mM EDTA, 20 mM Tris pH8 and 500 mM NaCl). Subsequently, the immunoprecipitated samples were eluted with 25 µl of 2x SDS sample buffer (See Western blotting). Standard Western blotting procedures were followed using anti-GRα, anti-FBXW7α and anti-GAPDH antibodies. For data representation see Figure legends.

### Immunofluorescence, co-localization and proximity ligation assay (PLA)

Following seeding, transfection with hGRwt or hGRdim for immunofluorescence and co-localization studies or GFP-hGRwt or GFP-hGRdim for PLA, cells were re-plated at 3 × 10^4^ cells/well into 8-well chambers (Nunc Lab-Tek Chamber Coverglass (TM) System) and treated as indicated in Figure legends. After treatment, cells were washed 3-times with warmed PBS, fixed, using 4% paraformaldehyde, and permeabilized, using 0.02% Triton-X. After permeabilization, cells were blocked (5% PBS-BSA for 1 hour), washed 3-times with 1.5% PBS-BSA for 5 minutes/wash and incubated for one hour with primary antibodies. To visualize GRα mouse anti-GRα antibody (ab2768, Abcam) followed by goat Alexa Fluor 594 anti-mouse IgG (Abcam) was used, while to visualize FBXW7α, rabbit anti-FBXW7α antibody (ab109617, Abcam) followed by goat Alexa Fluor 488 anti-rabbit IgG (Abcam) was used. For visualization of nuclei, Hoechst 33258 stain was used. The LSM780 confocal microscope with ELRYA PS1 super-resolution platform (Zeiss, Germany) and a LCI “Plan-Apochromat” 63×/1.4 Oil DIC objective was used for image acquisition. The microscope is equipped with a GaAsP detector, for signal collection. The 405 nm, 488 nm and 561 nm lasers, with their appropriate beam splitters (MB405 and MBS488/561) where used for exciting of the three respective fluorophores, while the signal detection for Alexa488 was from 490 to 552 nm, for Alexa594 from 611 to 733 nm and for DAPI from 410 to 473 nm. Moreover, the laser power and detection gains were optimized to prevent ‘bleed through’ and the image resolution was set at 1024 × 1024. Z-stacks were acquired and presented as maximum intensity projections. For data presentation, see Figure legends.

For co-localization of GRα and FBXW7α confocal images acquired following immunofluorescence were analysed using the powerful ZENN 2012 software, which displays the co-localization of two proteins, using white pixels containing both red and green intensities, in a 2D-scattergram, an example of which is detailed in Costes *et al*.^84^. Optimal thresholds for each channel were determined and the region where both individual channels were above their respective thresholds, was defined as the co-localization region^84^. For the quantification of the co-localization signal, the weighted co-localization coefficients were determined, using the defined thresholds and the ZENN 2012 software analysis to rank the pixel intensities in each channel, and plotted. For data presentation, see Figure legends.

For the proximity ligation assay (PLA), the immunofluorescence protocol above was followed up to primary antibody incubation with anti-GRα and anti-FBXW7α, after which the Duolink (Sigma-Aldrich) proximity ligation assay (PLA) was conducted as detailed by the manufacturer. For nuclei visualization Hoechst 33258 stain was used. For visualization, the LSM780 confocal microscope was used as described above. Briefly the three fluorophores were excited using the 405 nm, 488 nm and 561 nm lasers and the signal detected for the Duolink amplification signal from 611 to 733 nm, GFP from 499 to 561 nm and DAPI from 414 to 522 nm. Cells were selected for GFP-signal and the PLA signal, fluorescent red spots, quantified using IMAGEJ software and normalized to the GFP signal. For data presentation, see Figure legends.

### Statistical analysis

Statistical analysis throughout this study was conducted using the GraphPad Prism software, version 5. Specifically, one-way ANOVA followed by Tukey’s (comparing all means to every other mean) or Dunnett’s (comparing mean to a control mean) multiple comparisons post-test or two-way ANOVA followed by Bonferroni’s multiple comparison post-test (comparing all means to every other mean) were used for comparisons of multiple groups with equal variances as indicated in Figure legends. ANOVA was performed on logarithmically transformed data. To compare two groups we used the unpaired two-tailed Student’s t-test with Welch’s correction. Alpha was set to 0.05 for all analysis. Values are means ± SEM with number of biological repeats indicated in all figure legends. *P < 0.05, **P < 0.01, ***P < 0.001, ^#^P < 0.05 or ^##^P < 0.01, ^###^P < 0.001, for comparisons as indicated. In addition, different letters were used to distinguish statistically different groups, where the same letter represents no significant difference between values whilst letters, which are different are significantly different from each other P < 0.05.

## Electronic supplementary material

Supplementary Information

## Data Availability

All data generated or analysed during this study are included in this published article (and its Supplementary Information files).
