# Supplementary material for: Novel role for receptor dimerization in post-translational processing and turnover of the GRα
Source: Sci Rep. 2018 Sep 24;8:14266. doi: 10.1038/s41598-018-32440-z (PMC6155283; doi:10.1038/s41598-018-32440-z)
Supplement: Supplementary file 1 — Supplementary Information [file 41598_2018_32440_MOESM1_ESM.pdf]

Supplementary Figure File

Novel role for receptor dimerization in post-translational processing and turnover of the GRα

Legh Wilkinson[1], Nicolette Verhoog[1] and Ann Louw[1*]

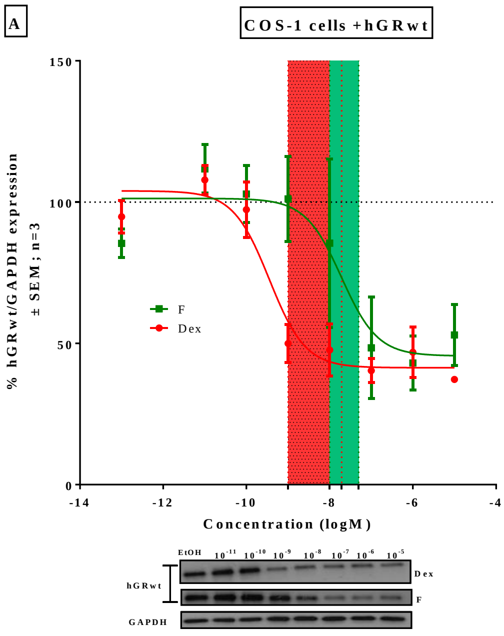

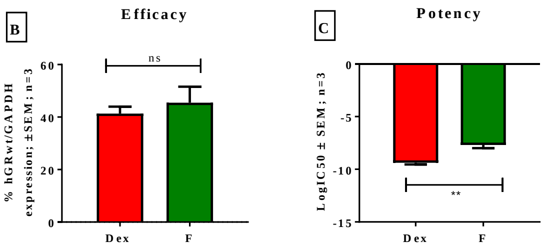

D

	hGRwt	
	Dex	F
	AVG ± SEM	AVG ± SEM
**Efficacy (%)**	41.3 ± 2.6	45.5 ± 6.2
**% hGRwt reduction**	58.7	54.5
**LogIC50**	-9.5 ± 0.2	-7.7 ± 0.3
**IC_{50} (nM)**	0.4	20

Supplementary Figure S1: hGRwt protein turnover is ligand and dose-dependent. COS-1 cells were seeded into a 24 well plate (5 x 10^4 cells/well) and transiently transfected the next day with hGRwt. Following 24 hours incubation, cells were treated with either solvent (EtOH) or varying concentrations (10^{-11} M to 10^{-5}M) of Dex and F for 24 hours. Thereafter, hGRwt protein expression was assessed by Western blotting where GAPDH was probed to ensure equal protein loading. The Western blot shown (A, inset) is representative of three independent experiments. For quantification (A), the intensity of the hGRwt and GAPDH bands was determined using UNSCANIT, the hGRwt expression was then normalized to GAPDH expression and expressed as a percentage (average ± SEM) of hGRwt expression in the presence of solvent (EtOH), which was set at 100% (dotted line). Physiological concentrations of Dex (red; 1-20 nM) and F (green; 10-50 nM) are indicated by shaded areas. Efficacy (B and D) and potency (C and D) of GCs for hGRwt down-regulation were determined. For statistical analysis, an unpaired two-tailed t-test with Welch's correction was used to evaluate the effects of ligands on the efficacy (ns, $P > 0.05$) and the potency (**, $P < 0.01$). Full-length blots are presented in Supplementary Figure S10.

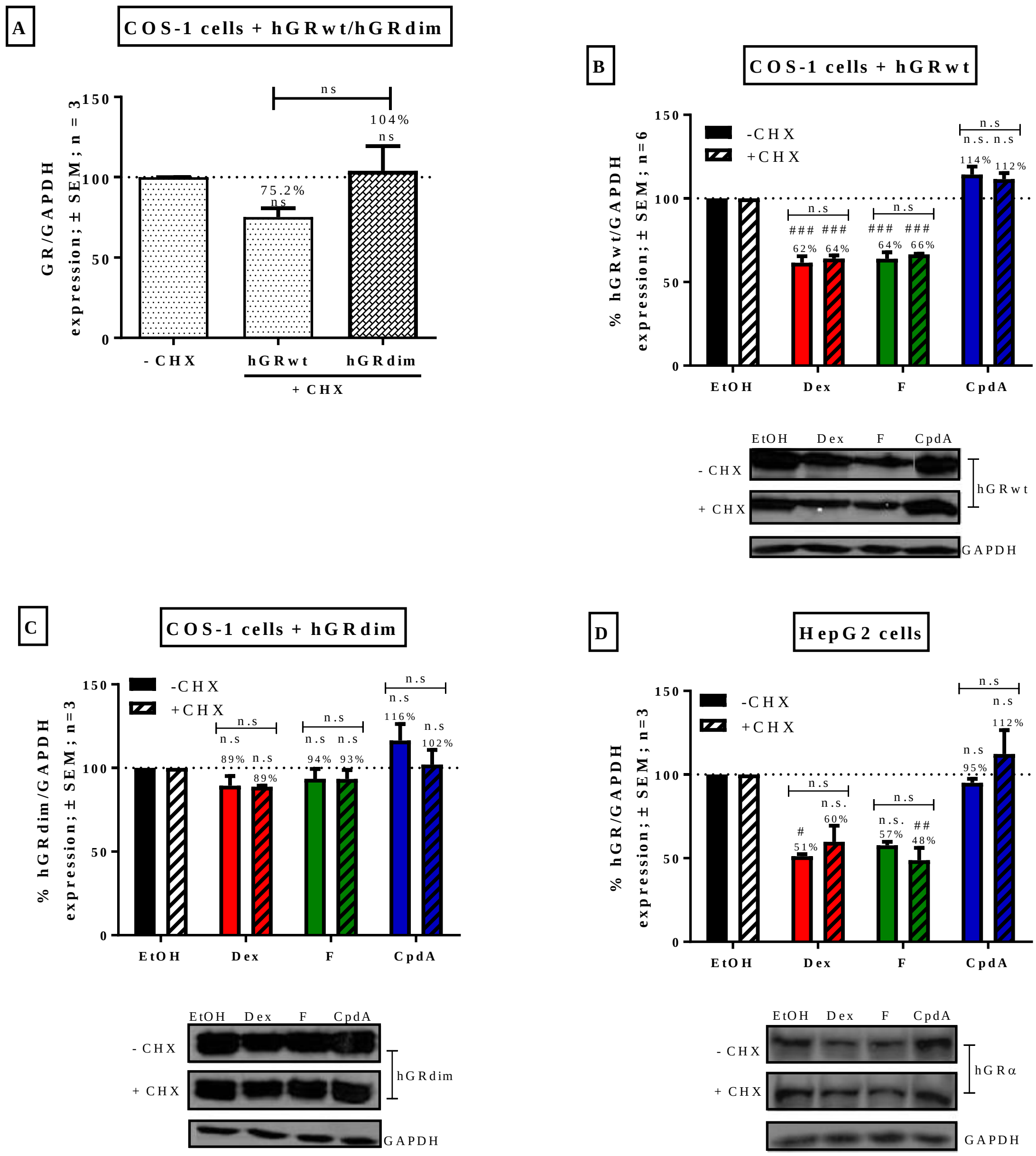

Supplementary Figure S2: Ligand-induced GRα protein turnover is unaffected by new protein synthesis. COS-1 cells were seeded in a 12 well plate (5 x 10^4 cells/well) and transiently transfected the next day with either hGRwt (A and B) or hGRdim (A and C). HepG2 cells with endogenous hGR were used in (D). Following 24 hours incubation, cells were treated with solvent (EtOH) or 1µM CHX for 1 hour and, in the absence (- CHX) or presence of CHX (+ CHX), with solvent (EtOH) or the compounds, Dex, F and CpdA (10^{-5}M), for a further 16 hours. GRα protein expression was assessed by Western blotting, where GAPDH was probed to ensure equal protein loading. The Western blots shown (B, C and D inset) are representative of three independent experiments. For quantification (A, B, C and D), the intensity of the GRα and GAPDH bands was determined using UNSCANIT and the GRα expression was then normalized to GAPDH expression expressed as a percentage (average ± SEM). Firstly, in the presence of solvent (EtOH), the effect of CHX (+ CHX) on unliganded GRα protein turnover was investigated (A) and compared to GRα of expression in the absence of CHX (- CHX). Thereafter, the effect of CHX (+ CHX) on the extent of hGRwt (B), hGRdim (C) and endogenous hGRα (D) ligand-induced turnover was investigated. The dotted line, in all graphs, represents GRα expression in the absence (- CHX) and/or presence of CHX (+ CHX) and in the presence of solvent (EtOH) and is set at 100%. To analyse the effects of the ligands on GRα expression in the absence (- CHX) and presence (+ CHX) of CHX, statistical analysis was conducted on logarithmically transformed data using a two-way ANOVA with a Bonferroni's multiple comparisons test comparing GRα expression post Dex, F and CpdA-treatment to the solvent (EtOH) (ns, $P > 0.05$, $^{\#}$, $P < 0.05$, $^{\#\#\#}$, $P < 0.001$) (B, C and D). Full-length blots are presented in Supplementary Figure S11.

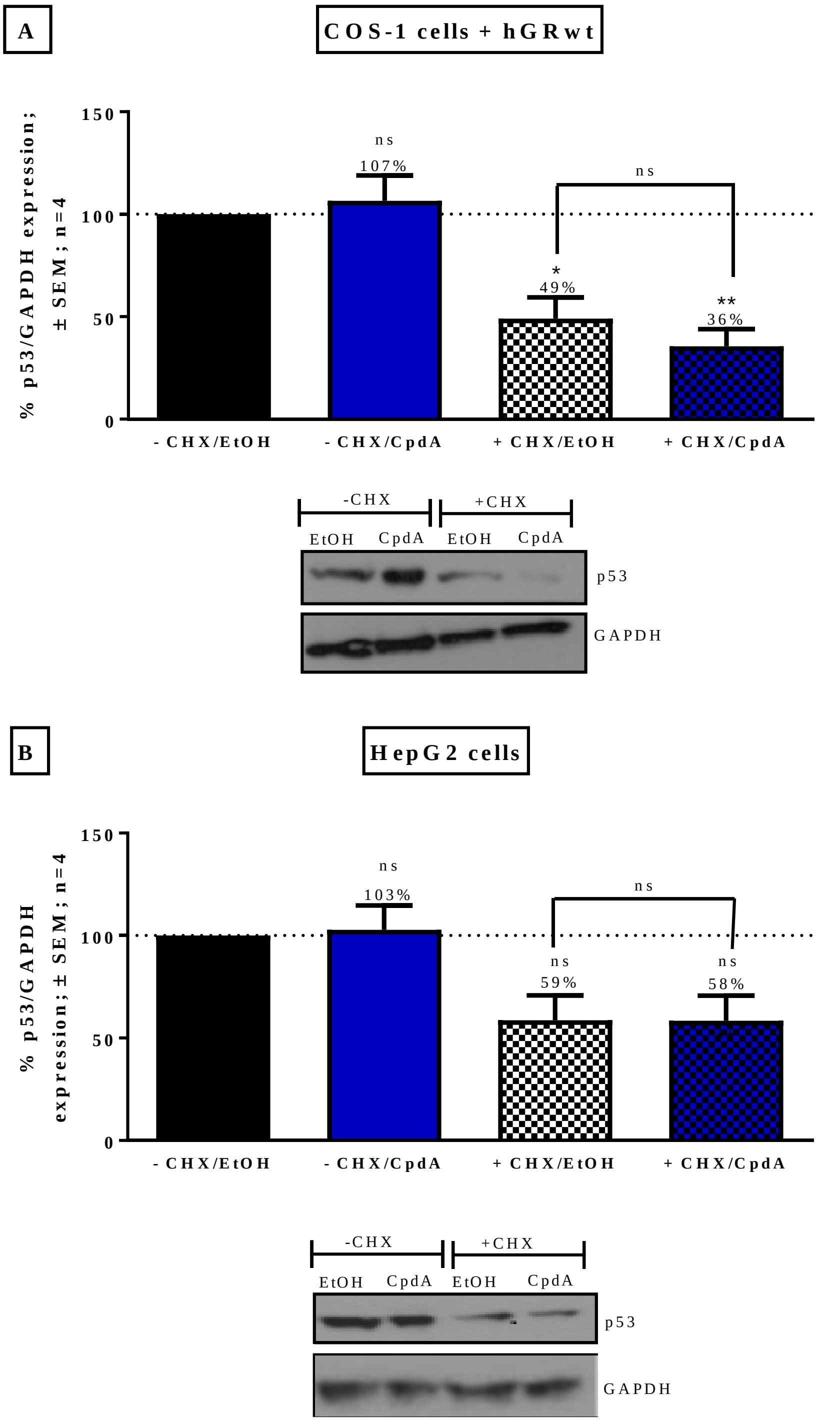

Supplementary Figure S3: CpdA treatment does not affect proteasome function. COS-1 (A) and HepG2 cells (B) were seeded in a 12 well plate (5 x 10^4 cells/well). Following 24 hours incubation, cells were treated with EtOH (solvent) or 1µM CHX for 1 hour and, in the absence (- CHX) or presence of CHX (+ CHX), with EtOH (solvent) or CpdA (10^{-5}M) for 16 hours. The p53 protein expression was assessed using Western blotting, where GAPDH was probed to ensure equal protein loading. The Western blots shown below the graphs (A and B inset) are representative of four independent experiments. For quantification, the intensity of the p53 and GAPDH bands was determined using UNSCANIT and subsequently the p53 expression was normalized to GAPDH expression and expressed as a percentage (average ± SEM) of p53 expression in the presence of solvent (EtOH), which is set at 100% (dotted line). To demonstrate the effects of CHX and CpdA on p53 expression, statistical analysis was conducted on logarithmically transformed data using a one-way ANOVA with a Tukey's multiple comparisons post-test (ns, $P > 0.05$, *, $P < 0.05$, **, $P < 0.01$). Full-length blots are presented in Supplementary Figure S12.

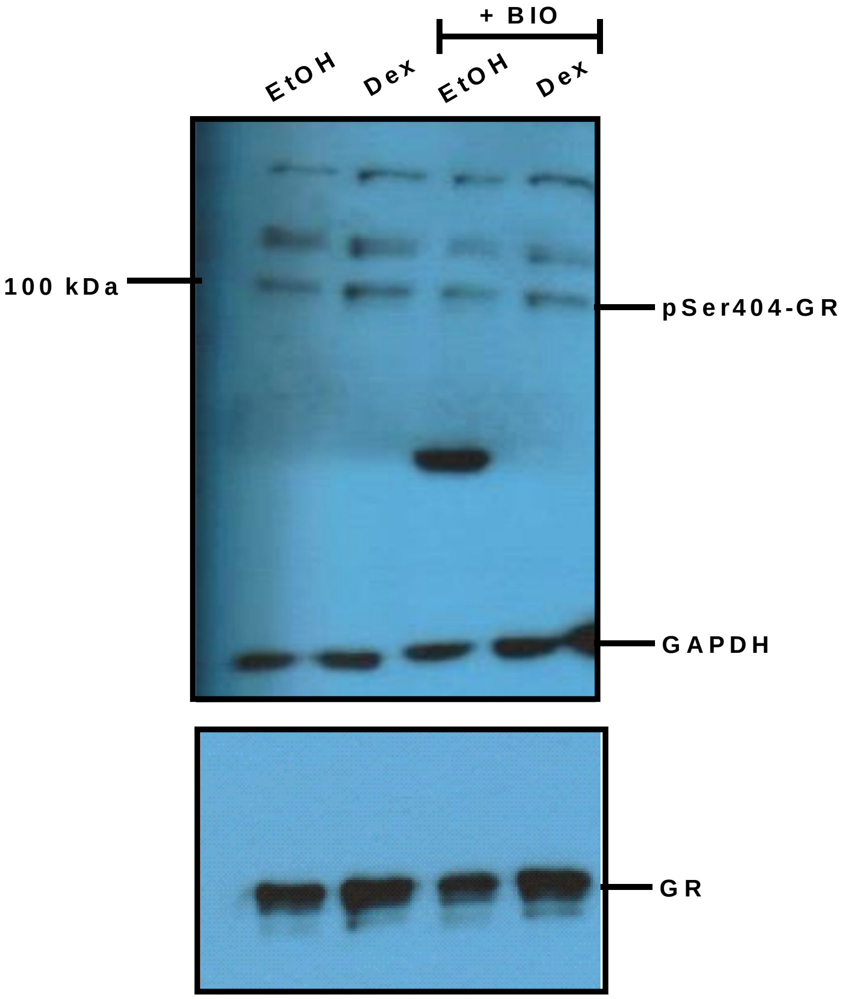

Supplementary Figure S4: Inhibition of the Dex mediated hyper-phosphorylation at S404, using the GSK3β inhibitor, BIO. HepG2 cells were treated with 5μM BIO (GSK3β inhibitor) for 1 hour prior to treatment with DEX (10^{-5}M). pS404-GR levels were detected using Western blotting. Blots were stripped and re-probed for total GRα protein content.

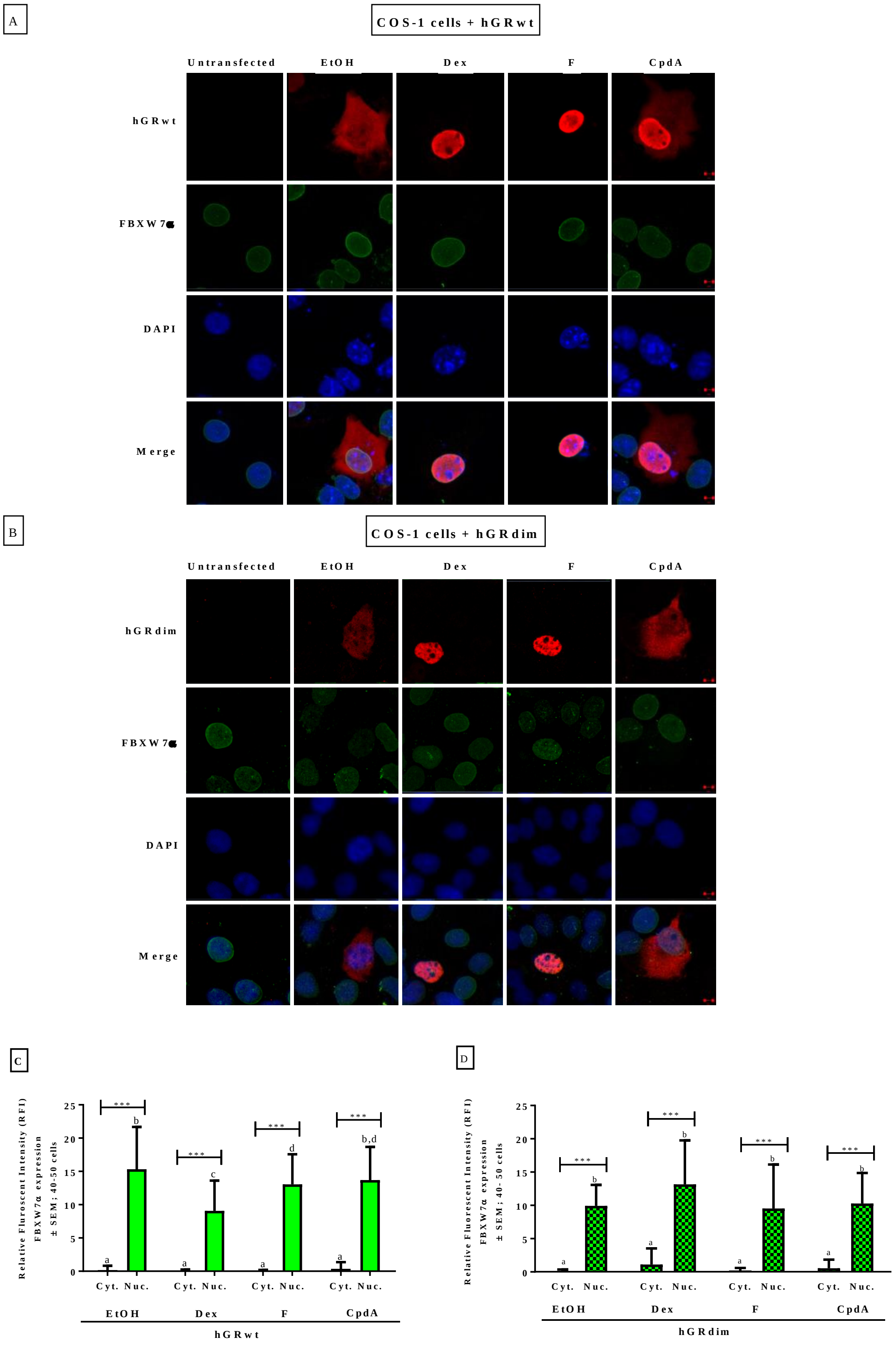

Supplementary Figure S5: Ligand-dependent subcellular localization of GRα and FBXW7α. COS-1 cells were seeded into a 10 cm dish (1 x 10^6 cells) and transiently transfected with either hGRwt or hGRdim. Following 24 hours incubation, cells were re-plated and treated with solvent (EtOH), Dex, F or CpdA (10^{-5}M) for 3 hours. Thereafter, cells were fixed, permeabilized, and immunofluorescence conducted, with antibodies specific for GRα

and FBXW7α. Cells were then imaged using a confocal microscope. A representative image illustrates the individual subcellular localization of (A) hGRwt or (B) hGRdim (red, first row) and (A and B) FBXW7α (green, second row), as well as the position of the cell's nucleus (blue DAPI stain, third row) with the merge representing an overlay of all three channels (red, green and blue). For the quantification of the subcellular localisation of FBXW7α in cells transfected with (C) hGRwt or (D) hGRdim the relative fluorescence intensity (RFI) of the green (FBXW7α) pixels was calculated for individual cells by selecting regions of interest (ROI), and plotted. Statistical analysis for FBXW7α subcellular localisation for cells transfected with hGRwt (C) or hGRdim (D) was conducted on logarithmically transformed data using a two-way ANOVA followed by a Bonferroni multiple comparisons post-test comparing experimental values to solvent's (EtOH) cytoplasm (for a,b, c and d, letters that are the same represent no significant difference between values whilst letters, which are different are significantly different from each other $P < 0.05$) or comparing the cytoplasmic and nuclear expression of FBXW7α within a treatment group (ns, $P > 0.05$ and ***, $P < 0.001$).

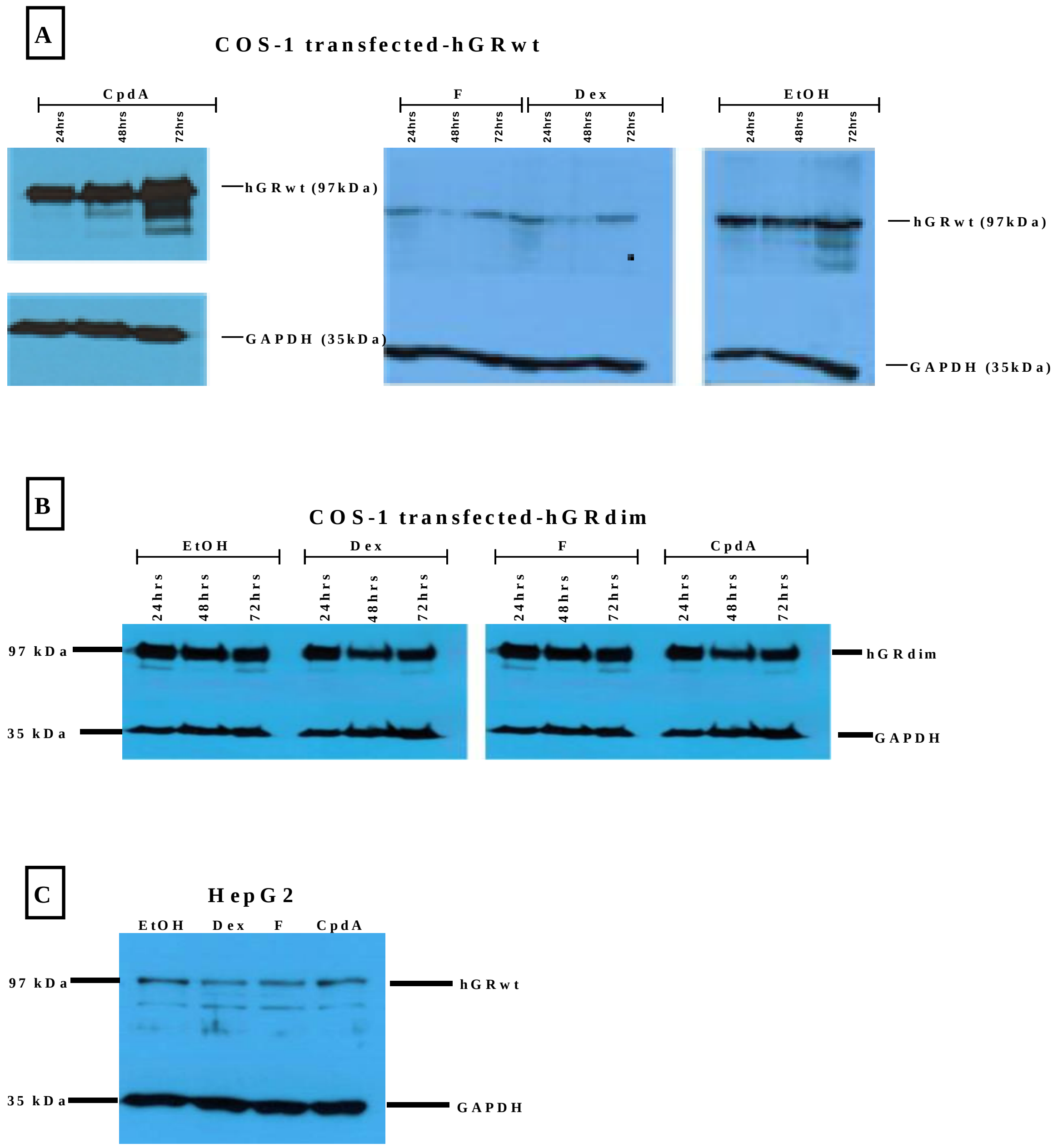

Supplementary Figure S6: Full-length blots pertaining to Figure 2.

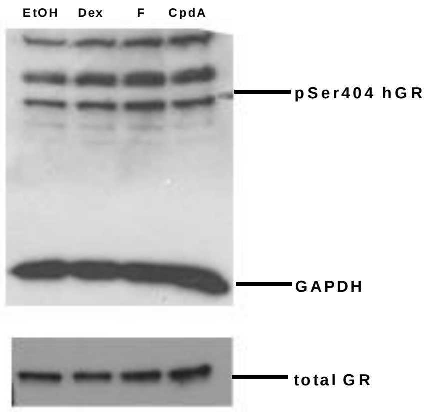

Supplementary Figure S7: Full-length blots pertaining to Figure 4A.

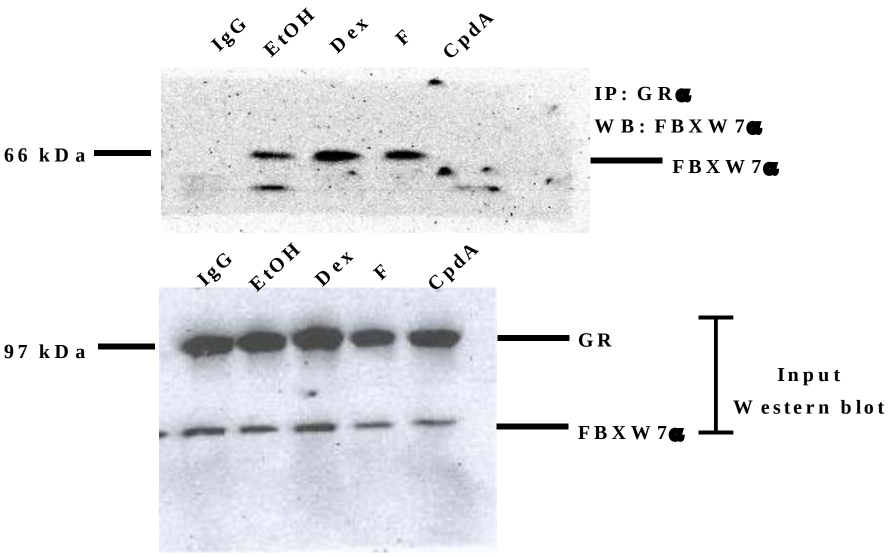

Supplementary Figure S8: Full-length blots pertaining to Figure 6.

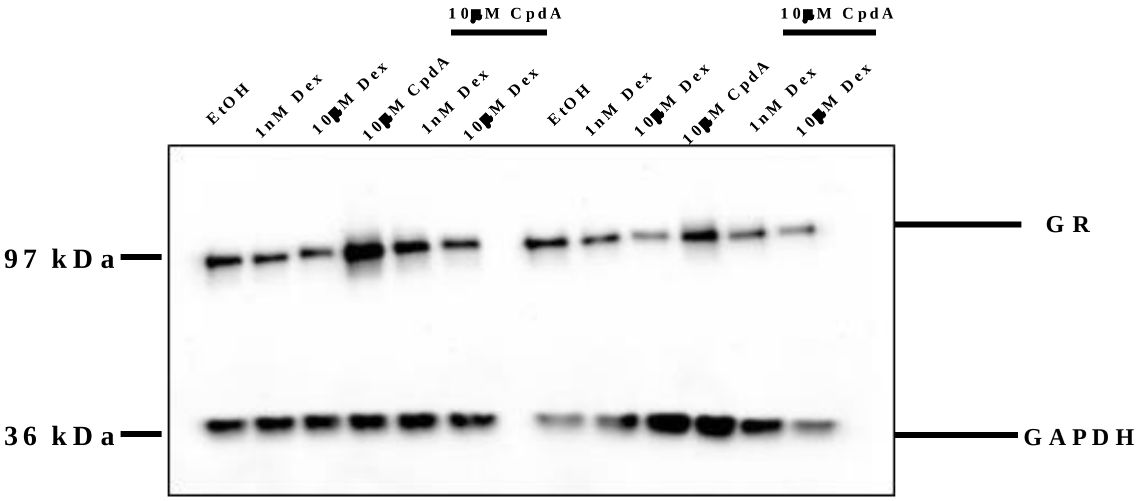

Supplementary Figure S9: Full-length blots pertaining to Figure 7.

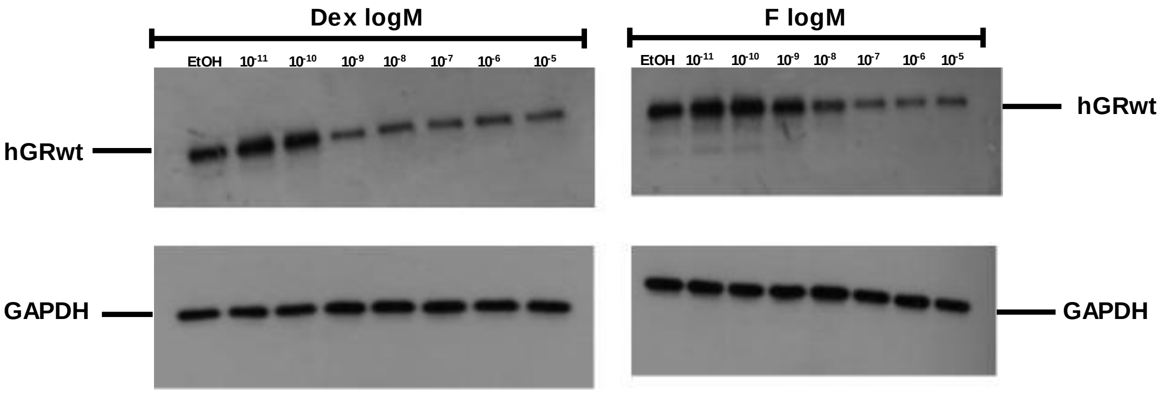

Supplementary Figure S10: Full-length blots pertaining to Supplementary Figure S1.

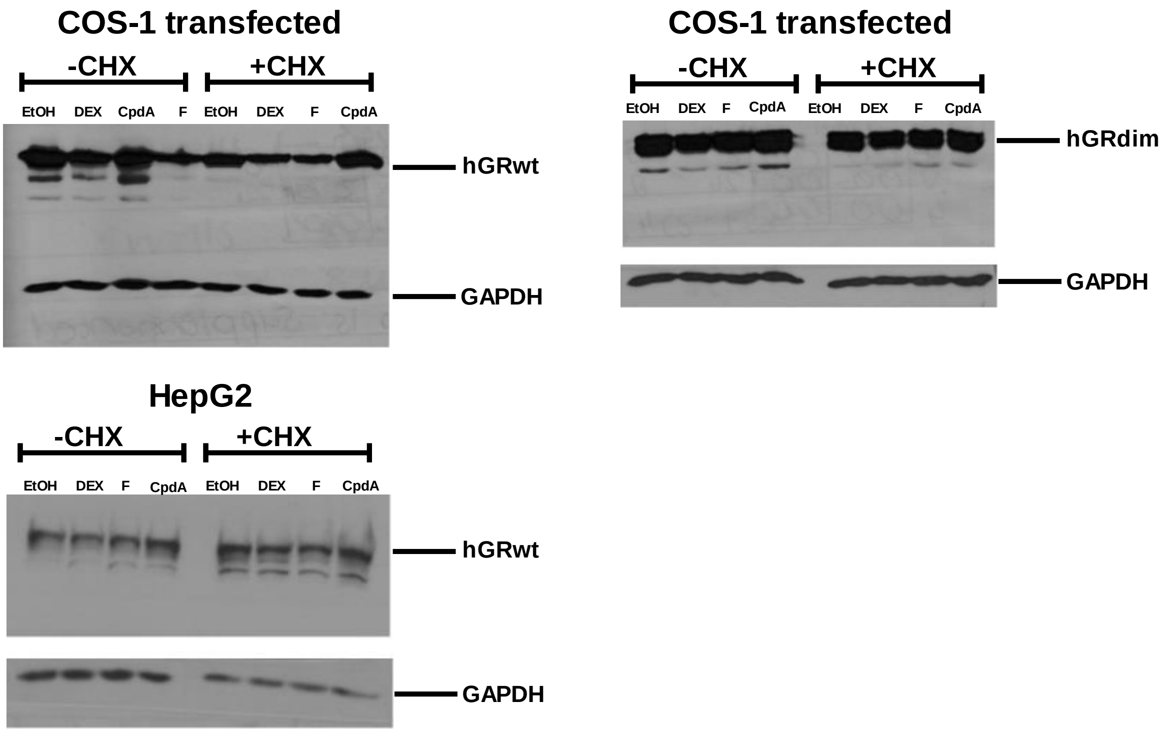

Supplementary Figure S11: Full-length blots pertaining to Supplementary Figure S2.

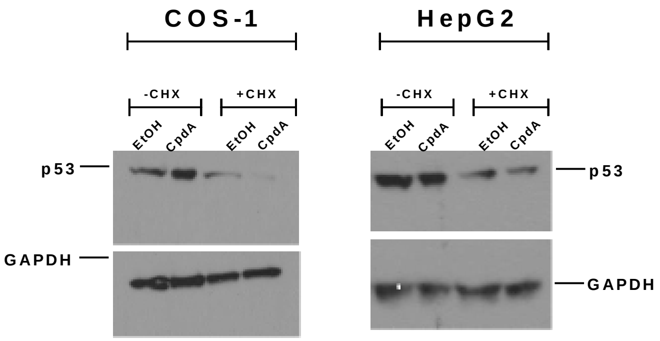

Supplementary Figure S12: Full-length blots pertaining to Supplementary Figure S3.
